# A systematic literature review on the effects of mycotoxin exposure on insects and on mycotoxin accumulation and biotransformation

**DOI:** 10.1007/s12550-021-00441-z

**Published:** 2021-10-07

**Authors:** K. Niermans, A.M. Meyer, E.F. Hoek-van den Hil, J.J.A. van Loon, H.J. van der Fels-Klerx

**Affiliations:** 1 Department of Plant Sciences, Laboratory of Entomology, Wageningen University, Wageningen, The Netherlands; 2 Wageningen Food Safety Research, Akkermaalsbos 2, 6708 WB Wageningen, The Netherlands

**Keywords:** Contaminant, Food safety, Remediation, Circular economy, Development, Metabolization

## Abstract

Novel protein sources for animal feed are needed, and the use of insects as feed ingredient is explored. The insect production sector offers opportunities for a circular and sustainable approach to feed production by upgrading waste or side streams into high-quality proteins. However, potential food or feed safety issues should be studied in advance. Mycotoxins, such as aflatoxin B1, are natural contaminants commonly found in agricultural crops and have proven to be detrimental to the agricultural industry, livestock, and human health. This systematic review aims to provide a comprehensive overview of the published evidence on effects of mycotoxin exposure on insect growth and survival, mycotoxin accumulation within the insect body, and metabolization of various mycotoxins by insects. The review includes 54 scientific articles published in the past 55 years, in total covering 32 insect species. The main findings are the following: (1) Insects of the order Coleoptera show lower mortality after exposure to aflatoxin B1 when compared to Lepidoptera and Diptera; (2) effects of mycotoxins on larval growth and survival are less detrimental in later larval stages; (3) accumulation of mycotoxins was low in most insect species; (4) mycotoxins are metabolized within the insect body, the degree of which depends on the particular mycotoxin and insect species; (5) cytochrome P450s are the main family of enzymes involved in biotransformation of mycotoxins in some insect species. Results of this review support an optimistic outlook for the use of mycotoxin-contaminated waste streams as substrate for insect rearing.

**Supplementary Information:**

The online version contains supplementary material available at 10.1007/s12550-021-00441-z.

## Introduction

With the expected growth of the human population, an increase in food and feed production is required and the use of insects as a novel suitable feed source of animal proteins is explored. Opportunities for a circular and sustainable approach to feed production are offered by the insect production sector. Traditionally, more than 2000 species of insects are consumed (Jongema 2017), of which most in tropical countries. Beetles (Coleoptera), butterfly and moth larvae (Lepidoptera), and ants, bees, and wasps (Hymenoptera) are consumed most commonly, followed by crickets, grasshoppers, and locusts (Orthoptera) (Van Huis et al. 2013). However, in Europe, the consumption of insects is still considered novel. Besides increasing the use of insects as food, their use in the feed sector provides interesting opportunities. By using waste or side streams as substrates for insect rearing low-quality streams can be upgraded into high-quality proteins. But possible issues regarding food or feed safety should be studied beforehand. Serval classes of contaminants could possibly be present in waste or side streams of interest. When using these waste or side streams as a substrate to rear insects as food and/or feed, it is important to know whether these insects accumulate the possibly present contaminants in their bodies and therefore become a source of contaminants themselves. Waste or side streams can originate from a variety of sources. This wide variety of available waste or side streams could also result in contamination by heavy metals, veterinarian drugs and hormones, pesticides, dioxins, dioxin-like polychlorinated biphenyls, and polyaromatic hydrocarbons, among others. Additionally, an example of a group of contaminants commonly found in nature and in agriculture are mycotoxins (van der Fels-Klerx et al. 2018). Mycotoxins are a chemically diverse group of low-molecular weight secondary metabolites produced by fungi, mainly *Aspergillus* spp., *Fusarium* spp., and *Penicillium* spp. Mycotoxins can cause a variety of adverse effects on human and animal health (Hussein and Brasel 2001) and are commonly found in seeds, nuts, and ears of crops (Agriopoulou et al. 2020). Therefore, waste or side streams consisting of agricultural materials, for example, restaurant waste or brewery spent grains, could be contaminated with these mycotoxins and be fed to insects when used as substrates for insect rearing. As mycotoxins are detrimental to both human and animal health, the European Union (EU) has set maximum levels (MLs) for the presence of certain mycotoxins in food and food commodities in Commission Regulation (EC) No. 1881/2006 (EC 2006a). MLs and advised guidance values for feed materials and complementary and complete feeding stuffs in Directive 2002/32/EC and Commission Recommendation 2006/576/EC (EC 2006b; EU 2002). Table 1 gives an overview of the range of MLs and advised guidance values set for feed and food materials. For a detailed overview of which ML or guidance value is set for a specific product intended as animal feed or as food, we direct the reader to the legal documents themselves. Data on the occurrence of mycotoxins in raw materials used for animal feed production (maize, wheat, barley and soybean) from 100 countries, collected in the past 10 years, showed that mycotoxin concentrations mostly complied with the ML or guidance values set for animal feed in the EU (Gruber-Dorninger et al. 2019). However, the percentage of samples exceeding the ML or guidance values varied between 2.4–7.4% for AFB_1_, 4.8–13.0% for ZEN, 4.3–21.5% for DON, 0.2–0.9% for OTA, and 0.0–3.3% for FB_1_ + FB_2_. This study showed that mycotoxin presence in feed and related commodities greatly varies from year-to-year and varies between regions of Europe, being highly affected by environmental conditions and agricultural practices (Gruber-Dorninger et al. 2019). Climate change and bad storage conditions may result in elevated levels of mycotoxins in plants and crops (e.g., maize) on which insects feed (Gruber-Dorninger et al. 2019; Medina et al. 2017). Some insect species have a degree of tolerance to particular mycotoxins and — in general — insects seem to be able to grow on plant-derived (waste) streams which contain mycotoxins (Bosch et al. 2017; Leni et al. 2019; Niu et al. 2009; Ochoa Sanabria et al. 2019). When comparing the initially present mycotoxin concentration with the concentration found in the residual feed material and the larvae, a portion of the ingested mycotoxins could not be recovered (Leni et al. 2019; Schrögel and Wätjen 2019) pointing at a need to further investigate the fate of mycotoxins. These unrecovered mycotoxin fractions could indicate the formation of mycotoxin metabolites (Berenbaum et al. 2021), or adducts (with protein or DNA), of which some could be unknown. Possible metabolites and modified forms of mycotoxins formed after ingestion of mycotoxins by insects may still be toxic (e.g., formation of a more toxic metabolite) to animals or humans (EFSA 2016), indicating the necessity for more information on the metabolism of mycotoxins by insects especially if intended for use as feed and food.

**Table 1:** Overview of the range of MLs and advised guidance values set for products intended for animal and human consumption

Mycotoxin	Maximum levels in µg/kg	Guidance values in µg/kg
**All feed materials and complementary and complete feeding stuffs**		
AFB_1_	5–20^1^	
DON		900–12,000^2^
Sum of FB_1_ and FB_2_		5000–60,000^2^
OTA		50–250^2^
ZEN		100–3000^2^
**Foodstuffs**		
Sum of AFB_1_, B_2_, G_1_, and G_2_	4–15^3^	
DON	200–1750^3^	
Sum of FB_1_ and FB_2_	200–2000^3^	
OTA	0.50–10^3^	
Patulin	10–50^3^	
ZEN	20–200^3^	

^1^Directive 2002/32/EC

^2^Commission Recommendation 2006/576/EC

^3^Commission Regulation (EC) No 1881/2006

Over the past years, the topic of insects as food and feed has received increasing attention and has been elaborately discussed by Arnold van Huis (Van Huis 2016, 2020; Van Huis et al. 2013). Also, the topics of sustainability in insect rearing (Van Huis and Oonincx 2017), consumer acceptance (Kauppi et al. 2019), profitability (Niyonsaba et al. 2021), and the circular business model perspective (Madau et al. 2020) have been extensively discussed in recent reviews. To date, the available data on possible accumulation of mycotoxins in insects and the possible metabolization of these toxins by insects, as well as the possible data gaps, are fragmented across disciplines and no clear overview is available. Therefore, the aim of this study was to obtain a comprehensive overview of the available information on the effects of mycotoxin exposure on growth and survival of insects, possible accumulation of mycotoxins in insects, and the possible bio-transformation of mycotoxins by insects. To this end, a systematic review was done covering all insect species, but with a particular focus on species used for feed and food. This comparative approach may allow extrapolation to a wider range of insect species that may be used for feed and food production in the future.

## Methods

Three different bibliographic databases (i.e., PubMed, CAB Abstracts, and Scopus) were used to retrieve peer-reviewed studies published in the English language from 1950 up to and including 2020. Search strings were defined beforehand and were divided into two parts. The general keywords added in both sets of search strings were as follows: *larva(e)*, *larval*, *insect(a)*, *insects*, *mycotoxin(s)*, deoxynivalenol*, enniatin*, beauvericin*, nivalenol*, aflatoxin*, zearalenone*, fumonisin*, and ochratoxin*. The first search focused on survival and development of insects and included the following additional keywords: *life cycle stage(s)*, life*cycle, *life stages*, *biomass*, reprod* fitness*, grow*, develop*, *mortality*, weigh*, pupat*, and surviv*. The second search focused on accumulation and transformation of mycotoxins and contained the following additional keywords: metabolism*, *convert*, *conversion*, *breakdown*, degrad*, accumulat*, conjugat*, absorb*, excret*, distribut*, and adme*. For all search strings used, it was ensured that respective plural forms as well as related words (synonyms) were covered. The collected articles were stored in an EndNote library after which duplicates were removed. Then, the articles were screened for their relevance by using a priori determined exclusion criteria. Exclusion criteria used included the following: non-English articles, non-research articles, review articles, no full text available (via the WUR library), not focusing on insects (i.e., class Insecta), studies in insect cell lines, not focusing on mycotoxins, and focusing on pest management. The snowballing technique was used to identify other relevant studies from reference lists of the articles found.

Data reported in the relevant articles were extracted and synthesized to provide an overview of effects of mycotoxins on insect mortality and growth, on accumulation of mycotoxins, and on conversion of mycotoxins. Throughout the remaining part of this review, metabolism is defined as the process of biotransformation facilitated by enzymes to create polar compounds which are more easily excretable. The words metabolism, conversion, and biotransformation of mycotoxins will be used interchangeably to refer to metabolic processes which convert mycotoxins within insects into their metabolites or modified forms.

## Results

### Literature search

**Fig. 1:**
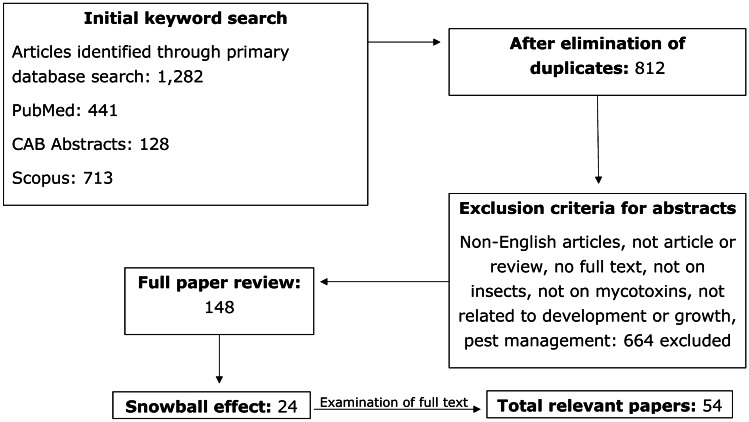
Overview of steps with number of articles of the systematic review process

The initial literature search yielded 1282 articles (Fig. 1). Following elimination of duplicates and screening of title, keywords, and abstract using the exclusion criteria, 148 potentially relevant papers were selected. An additional 24 potentially relevant papers were added in this step via the snowballing technique. After examination of the full texts, 52 papers were considered relevant. Additionally, two relevant papers which were published during the time this review was written were added. All study details and data from the final set of 54 research articles are presented in Supplementary Table S1, including the following: insect species, substrate used, exposure time of the insects, analytical method used, and mycotoxins (metabolites) analyzed.

Although all insect species were included in the review, most of the retrieved studies focused on species from the insect orders Diptera, Coleoptera, and Lepidoptera. The order Diptera includes fly species that can feed on a variety of organic residues and usually have a short life cycle. The Coleoptera are beetles and include known agricultural pests that can break down animal and plant debris. The Lepidoptera order includes butterflies and moths, the plant-feeding larvae of which are called caterpillars that can be detrimental to agriculture. The majority of currently considered edible insect species belong to the orders Coleoptera and Lepidoptera (Jongema 2017).

### Development and growth

#### Diptera

Developmental effects due to exposure of mycotoxins were observed mostly for larvae of *Hermetia illucens* L. and *Drosophila melanogaster* Meigen within the order of Diptera. Larval weight gain of *H. illucens* consuming a substrate contaminated with 4.600 µg/kg DON, 260 µg/kg OTA, 88 µg/kg AFB_1_, 17 µg/kg AFB_2_, 46 µg/kg AFG_2_, and 860 µg/kg ZEN was not significantly different as compared to the control treatment (Purschke et al. 2017). A significantly longer development time was observed in *Drosophila simulans* Sturtevant female larvae when exposed to 0.05 µg/kg OTA as compared to the control (Cao et al. 2019). Furthermore, as the concentration of AFB_1_ increased, more malformed adult (wings, leg and thorax) individuals of *D. melanogaster* were observed. Exposure to 800 µg/kg AFB_1_ resulted in 11% malformation as opposed to 7% for larvae exposed to 200 µg/kg AFB_1_ (Şişman 2006). Furthermore, feeding *D. melanogaster* (strain Oregon R) with 10,000 µg/kg AFB_1_ resulted in a doubling of larval and pupal development time (Kirk et al. 1971). Additionally, it has been shown that effects caused by mycotoxin exposure of *D. melanogaster* on growth can vary between both strains and larval stages. For instance, when 1st, 2nd, and 3rd instar larvae of strain A-11 of *D. melanogaster* were reared on 440 µg/kg AFB_1_, no significant growth effects were observed; however, 2nd instar larvae of strain A-9 raised on 440 µg/kg AFB_1_ developed into significantly smaller adults than their controls (Chinnici et al. 1979). In addition, *D. melanogaster* larvae fed with 200 µg/kg AFB_1_ led to a significantly smaller body length for larvae of the Florida-9 strain (Gunst et al. 1982).

#### Coleoptera

Growth effects caused by mycotoxin exposure were also observed in the order of Coleoptera. For example, developmental time of 1-day-old *Ahasverus advena* Waltl larvae was significantly longer when exposed to 2,000,000 µg/kg AFB_1_ (Zhao et al. 2018). Lifetime fecundity of *Tribolium confusum* Jacq. adults fed with 100,000 µg/kg T-2 for 120 days was not affected; however, this exposure resulted in a much higher egg production in the first 60 days, followed by a lower egg count in the last 60 days (Wright et al. 1976). Nonetheless, most available studies focused on the effect of mycotoxin exposure on larval weight. *Alphitobius diaperinus* Panzer larvae fed with 1300 µg/kg OTA had a significantly lower weight when compared to larvae fed with 1700 µg/kg OTA and the control (Camenzuli et al. 2018). Also, a lower weight was observed for *Zophobas atratus* Fabr*.* larvae fed on 500 µg/kg T-2 as compared to their control (Van Broekhoven et al. 2014). Most studies used *Tenebrio molitor* L. larvae and showed, for example, that exposure to 450,000 µg/kg FB_1_ resulted in a significantly lower weight in *T. molitor* larvae after 28 days (Abado-Becognee et al. 1998). Furthermore, *T. molitor* larvae gained significantly less weight when exposed to wheat bran contaminated with 8000 µg/kg DON for 2 weeks and this effect became more pronounced at increasing concentrations (Janković-Tomanić et al. 2019). Contrarily, *T. molitor* larvae exposed to either 500 µg/kg OTA, 500 µg/kg T-2 (Van Broekhoven et al. 2014), or 204 µg/kg AFB_1_ (Bosch et al. 2017) gained more weight than their respective controls, whereas no significant difference in weight gain of *T. molitor* larvae exposed to 415 µg/kg AFB_1_ was observed (Bosch et al. 2017). Additionally, exposure to flour that was naturally contaminated with 2854 µg/kg DON and 602.3 µg/kg ZEN resulted in a significantly increased larval weight (Niermans et al. 2019). *T. molitor* larvae fed with an artificial diet containing approx. 250 μg/kg T-2/HT-2 toxins gained 44% more weight than the control group fed a natural diet. Additionally, larval weight gain was significantly higher when fed with an artificially contaminated diet than when fed with naturally contaminated diets (Piacenza et al. 2020). In general, most studies focused on the effect of mycotoxin exposure on weight gain in *T. molitor* larvae.

#### Lepidoptera

Growth effects caused by mycotoxin exposure on Lepidoptera were reported in several studies. Firstly, 1st instar *Amyelois transitella* Walker larvae fed with 50,000 µg/kg AFB_1_ showed significantly lower pupation rates. Exposure of 5th instars to the same concentration did not cause a significant decrease in pupation rate. No developmental effects were observed in 1st instar *Am. transitella* larvae after exposure of up to 5000 µg/kg OTA, whereas 1st instar *Helicoverpa zea* Boddie larval development was significantly inhibited by this concentration of OTA (Niu et al. 2009). Exposure of *Spodoptera frugiperda* Smith and *Hel. zea* larvae to 25,000 µg/kg verrucolgen, roseotoxin B, or penitrem A for 7 days resulted in a significantly lower weight gain for both species. Additionally, exposure of *Hel. zea* larvae to 250 µg/kg penitrem A also resulted in a significantly lower weight; this was not observed when the larvae were exposed to the same concentration of roseotoxin B (Dowd et al. 1988). Exposure to 25,000 µg/kg DON caused a significant growth retardation in *Hel. zea* larvae but not in *S. frugiperda* larvae. In contrast, exposure to 25,000 µg/kg T-2 resulted in a significantly lower weight of *S. frugiperda* larvae only. However, 25,000 µg/kg diacetoxyscirpenol caused a significant growth retardation in both species (Dowd 1990), whereas exposure to 250,000 µg/kg griseofulvin caused significant weight loss in both *Hel. zea* and *S. frugiperda* larvae (Dowd 1993). Similarly, no significant difference in weight of *Hel. zea* larvae was observed after exposure to 25,000 and 250,000 µg/kg fusaric acid for seven days (Dowd 1989). Paterson et al. (1990) reared *S. frugiperda* larvae on substrates containing either brevianamide A, brevianamide D, or OTA, each at 10,000 µg/kg, and observed a significant larval weight loss after 3 days of exposure to each of the three mycotoxins. Exposure to brevianamide D reduced larval weight more than brevianamide A (Paterson et al. 1990). In neonatal *Spodoptera exigua* Hübner larvae reared for 7 days on a semi-synthetic diet containing 15,000–90,000 µg/kg destruxin B resulted in significant decrease in growth with increasing concentration (Rizwan-Ul-Haq et al. 2009). Destruxins are produced by the documented insect pathogenic fungus *Metarhizium* spp., but they are not common contaminants of food or feed.

AFB_1_ and its metabolites caused effects on fecundity and hatchability in *Spodoptera littoralis* Boisduval larvae. Exposure to 2500 µg/kg AFB_1_ or 4000 µg/kg AFG_1_ caused significant retardation in the development of both larvae and pupae and significantly reduced the percentage of hatchability. Exposure to either 2000 µg/kg AFB_1_, 3000 µg/kg AFG_1_, or 4000 µg/kg AFB_2_ caused a significant reduction in the numbers of eggs laid (Sadek 1996). Tolerance of *Trichoplusia ni* Hübner larvae to AFB_1_ seems to increase with age. As an example, exposure of newly hatched larvae to a semi-synthetic wheat germ-based diet containing 200 µg/kg AFB_1_ resulted in a significant inhibition of larval growth after ten days, while no negative effects on growth and development were observed in 5-day-old-larvae exposed to the same concentration for 3 days. Additionally, exposure of seven-day-old larvae to 3000 µg/kg AFB_1_ significantly reduced pupation, while exposure of 10-day-old larvae to the same concentration did not affect pupation (Zeng et al. 2013). Ten-day-old *Corcyra cephalonica* larvae needed to be exposed to at least 1,000,000 µg AFB_1_/kg for 12 days to observe a significantly reduced growth (Hegde et al. 1967). In *Bombyx mori* L. larvae, oral administration of up to 16,000 µg/kg bassianolide decreased body weight with an increasing dose already after 2 days. Larvae exposed to 4000 µg/kg bassianolide weighed half of the control group; however, statistical significance was not calculated in this study (Kanaoka et al. 1978). Bassianolide is produced by the well-known insect pathogenic fungus *Beauveria bassiana*, but is not a common contaminant of food or feed. *Choristoneura fumiferana* Clemens larvae grown on *Picea glauca* branches infected with rugulosin-producing endocytes showed that the *Ch. fumiferana* larvae grown on infected trees containing 850 µg/kg rugulosin (geometric mean) were significantly smaller than the ones grown on uninfected trees (Miller et al. 2008). Sumarah et al. (2008) performed a similar experiment and observed a significant reduction of growth of *Ch. fumiferana* and *Lambdina fiscellaria* Guenée larvae after exposure to 13,650 and 27,125 µg/kg rugulosin, respectively. A significant reduction of the head capsule was observed in *Ch. fumiferana* larvae fed with 54,250 µg/kg dietary rugulosin and in *L. fiscellaria* larvae fed with 81,375 µg/kg dietary rugulosin. However, larval weight of *Zeiraphera canadensis* Mutuura & Freeman did not significantly differ when exposed to up to 81,375 µg/kg rugulosin, which was the highest concentration tested (Sumarah et al. 2008).

#### Other orders

*Periplaneta americana* L. (Blattodea) fed sucrose contaminated with 12,000 µg/kg AFB_1_ had a higher body weight (approximately 7%) as compared to the control (Llewellyn et al. 1976). However, *Oncopeltus fasciatus* Dallas (Hemiptera) were observed to have a significantly lower body length after feeding on 5000 µg/kg AFB_1_ at 20 °C, whereas no effect on body length was observed when exposed to the same concentration at 25 °C (Llewellyn et al. 1988).

### Mortality

#### Diptera

**Fig. 2:**
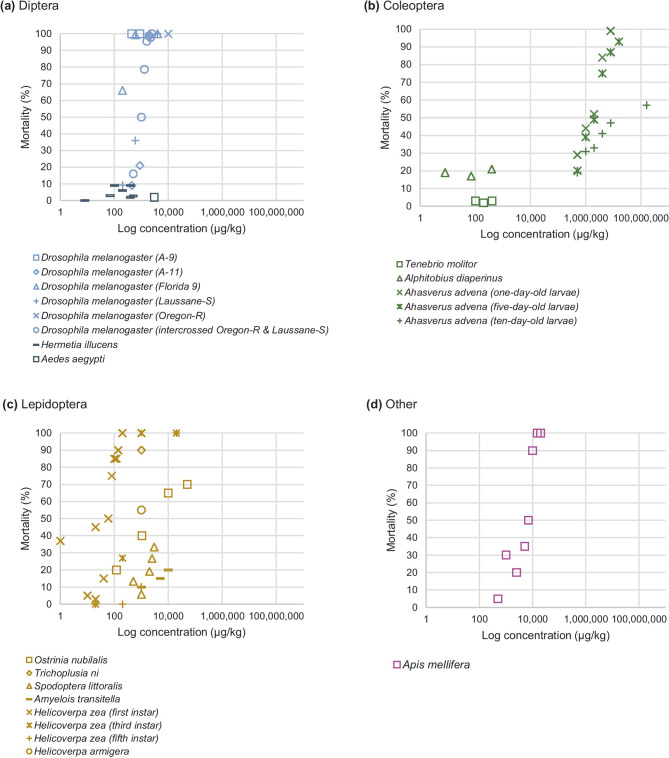
Mortality (%) caused by exposure to different doses of AFB_1_ (log scale) for 14 insect species belonging to four orders. Based on the studies of **a**: Camenzuli et al. (2018), Chinnici et al. (1979), Gunst et al. (1982), Kirk et al. (1971), Matsumura and Knight (1967), Meijer et al. (2019), Melone and Chinnici (1986); **b**: Bosch et al. (2017), Camenzuli et al. (2018), Zhao et al. (2018); **c**: Mencarelli et al. (2013), Niu et al. (2009), Sadek (1996), Zeng et al. (2006), Zeng et al. (2013); **d**: Niu et al. (2011). An overview of the data used is available in Table S2 of the Supplementary Materials. Figure is made in Excel

*Hermetia illucens* and *D. melanogaster* differed in tolerance to AFB_1_ exposure (Fig. 2a). When exposed to 1–500 µg AFB_1_/kg, < 30% mortality was observed in *H. illucens* (Bosch et al. 2017; Camenzuli et al. 2018; Meijer et al. 2019), while exposure to 440 µg AFB_1_/kg caused 100% mortality in *D. melanogaster* strain A-9, but only 9% mortality in *D. melanogaster* strain A-11 (Chinnici et al. 1979). Exposure to 200 µg AFB_1_/kg resulted in mortality of 66% and 9% in the *D. melanogaster* strains Florida 9 and Lausanne-S, respectively (Gunst et al. 1982). Interestingly, when intermated lines of *D. melanogaster* (strains Oregon-R and Lausanne-S) were exposed to AFB_1_ for multiple generations, a significantly enhanced resistance as compared to the control line was observed (Melone and Chinnici 1986). While most studies focused on AFB_1_, exposure of *H. illucens* larvae to DON in concentrations ranging from 630–3580 µg/kg resulted in mortality varying between 7 and 10% (Gulsunoglu et al. 2019). On the contrary, no significant difference in mortality was observed when exposing *H. illucens* larvae to a substrate that contained a mixture of DON (4600 µg/kg), 88 µg AFB_1_/kg, 17 µg AFB_2_/kg, 46 µg AFG_2_/kg, 260 µg OTA/kg, and 860 µg ZEN/kg (Purschke et al. 2017). Exposure of *H. illucens* larvae to spiked concentrations of AFB_1_ (390 µg/kg), DON (112,000 µg/kg), ZEN (13,000 µg/kg), and OTA (1700 µg/kg) caused ≤ 6% mortality (Camenzuli et al. 2018). Additionally, 2% mortality was observed in 4th instar *Aedes aegypti* L. larvae when exposed to 3000 µg AFB_1_/kg for 5 days (Matsumura and Knight 1967).

#### Coleoptera

In the order of Coleoptera, studies were performed on *T. molitor*, *Ah. advena*, *A. diaperinus*, *Z. atratus*, and *Tri. confusum*. Similar to the order of Diptera, differences in mycotoxin susceptibility were identified between Coleoptera species (Fig. 2b). Mortality in 1-day-old *Ah. advena* larvae caused by AFB_1_ seems to be dose-dependent and ranged from 29% when exposed to 500,000 µg AFB_1_/kg up to 99% when exposed to 8,000,000 µg AFB_1_/kg (Zhao et al. 2018). *T. molitor* larvae have overall proven to be quite tolerant to various mycotoxins and as shown in Fig. 2b — exposure to 415 µg AFB_1_/kg resulted in only 5% mortality (Bosch et al. 2017). Additionally, no significant effects on mortality were observed when *T. molitor* larvae were fed with contaminated diets containing 200–12,000 µg DON/kg, and exposure to substrates infested with 12,000 µg DON/kg led to 2% mortality (Ochoa Sanabria et al. 2019). A naturally contaminated diet containing 4900 µg DON/kg and another diet spiked with 8000 µg DON/kg (Van Broekhoven et al. 2017) also resulted in 2% mortality. In addition, *T. molitor* larvae fed with wheat containing 568–4588 µg DON/kg and 589–2283 µg ZEN/kg did not result in significant mortality (Niermans et al. 2019). Seven percent of mortality was observed in *T. molitor* larvae when fed with 128,000 µg T-2/kg (Davis and Schiefer 1982), but no mortality was observed upon exposure to 450,000 µg FB_1_/kg (Abado-Becognee et al. 1998). However, 100% mortality was observed in *T. molitor* larvae after exposure to 102,280 µg beauvericin/kg (*Beauveria bassiana* strain B13/I11) (Cito et al. 2016). It should be noted that beauvericin is produced by the well-known insect pathogenic fungus *Beauveria bassiana* and is a common contaminant of grains (EFSA 2014). Larvae fed with either an artificially contaminated diet or a naturally contaminated diet containing approximately 100 and 250 μg/kg total T-2 / HT-2 for 4 weeks resulted in an average mortality of 11% in *T. molitor* larvae considering all diets. Interestingly, when fed the artificially contaminated diet, 16% higher mortality was observed as compared to the naturally contaminated diet, and this effect seemed to be independent of the concentration (Piacenza et al. 2020). Camenzuli et al. (2018) exposed *A. diaperinus* larvae to 17 different treatments, including single spiked concentrations of AFB_1_ (8–390 µg/kg), DON (3900–112,000 µg/kg), ZEN (280–13,000 µg/kg), OTA (170–1700 µg/kg) and combined spiked mycotoxin concentrations of up to 100,000 µg/kg and found no significantly different mortality in these treatment groups as compared to the control group (Camenzuli et al. 2018). In another study, exposure to 500 µg/kg ZEN, OTA, and T-2 led to 10% mortality in *T. molitor* and *Z. atratus* larvae and 20% mortality for larvae of *A. diaperinus* (Van Broekhoven et al. 2014). A similar high mortality of 15% was observed in *Tri. confusum* larvae fed on 100,000 µg T-2/kg (Wright et al. 1976).

#### Lepidoptera

Most studies on the effects of mycotoxin exposure on insects are performed for species in the order Lepidoptera. Similar to the orders previously described, different degrees of susceptibility in the species of this order were observed (Fig. 2c) (Niu et al. 2009; Zeng et al. 2006). First instars of *Hel. Zea*, exposed to 1000 µg AFB_1_/kg, showed 100% mortality after 15 days, while also 100% mortality was observed in 3rd instars exposed to 20,000 µg AFB_1_/kg for 21 days (Zeng et al. 2006). Additionally, exposure to 1000 µg AFB_1_/kg resulted in a significant increase in mortality (40–55%) after 6–9 days in *Hel. armigera* larvae (Elzaki et al. 2019), 35–55% mortality after 8 days in *Hel. zea* larvae (Zeng et al. 2009), and 90% mortality after 10 days in *Tr. ni* larvae (Zeng et al. 2013). *S. littoralis* larvae fed with a diet containing AFB_1_ (500–3500 µg/kg), AFB_2_ (2000–4000 µg/kg), and AFG_1_ (1000–4000 µg/kg) until pupation showed a similar mortality across these treatments and a mortality increasing with higher doses (16–53%). Additionally, exposure to a combination of 3500 µg AFB_1_/kg and 75,000 µg kojic acid/kg resulted in an almost 9% increase in mortality compared to AFB_1_ exposure alone (Sadek 1996). *Ostrinia nubilalis* Hübner (European corn borer) larvae, specialized on corn (*Zea mays*), showed a high tolerance towards AFB_1_ exposure with a calculated median lethal concentration (LC_50_) of 2300 µg/kg diet (Mencarelli et al. 2013). The silkworm *B. mori* showed 100% mortality after 4 days of exposure to 15,614 µg AFB_1_/kg (Ohtomo et al. 1975). Similarly, oral administration of 12,000 µg bassianolide/kg also was observed to be lethal to *B. mori* larvae after an exposure of 6 to 8 days (Kanaoka et al. 1978). Paterson et al. (1987) exposed *S. littoralis* larvae to a variety of mycotoxins, all at a concentration of 10,000 µg/kg. Highest mortality was observed after exposure to penicillic acid (90%) and brevianamide A (78%). Exposure to viomellein, OTA, cyclopenol, and citrinin led to 30%, 40%, 26%, and 48% mortality, respectively, indicating a varying susceptibility of *S. littoralis* larvae to different types of mycotoxins (Paterson et al. 1987). Exposure of *Spodoptera litura* Fabr. to 88–264 µg destruxin/kg body weight (*Metarhizium anisopliae* M-10 isolate) caused 30–90% mortality, after 48 h. Destruxin obtained from a *Metarhizium anisopliae* M-19 isolate needed to be fed in nearly three times the doses to obtain the same percentage of mortality (Sree and Padmaja 2008). Exposure to 40,000–60,000 µg destruxin B/kg showed 7 to up to 30% mortality after 3 days, gradually increasing to 60–90% mortality after 8 days of exposure (Rizwan-Ul-Haq et al. 2009). Paterson et al. (1990) reared *S. frugiperda* larvae on substrates containing brevianamide A, brevianamide D and OTA up to a concentration of 10,000 µg/kg for 3 days and observed no mortality caused by brevianamide A and D. However, the observed mortality until pupation was considered significant for all treatments (Paterson et al. 1990). *Am. transitella* larvae seemed less sensitive to OTA exposure; concentrations of 1000–50,000 µg OTA/kg for 12 days showed no significant difference in mortality and resulted in a 10% mortality in *Hel. zea* after exposure to 1000 and 5000 µg OTA/kg after 10 days (Niu et al. 2009). For both *S. frugiperda* and *Hel. zea* larvae exposed to 25,000 µg/kg dihydroxyaflavinine and roseotoxin B, a significantly higher mortality than the control was observed, while after exposure to 2500–25,000 µg penitrem A/kg, a significant higher mortality (≥ 15%) was only observed in *Hel. zea* (Dowd et al. 1988).

#### Other orders

*O. fasciatus* larvae showed 100% mortality when exposed to 5000 µg AFB_1_/kg for 20 days, whereas a lower mortality was found after a shorter exposure time (Llewellyn et al. 1988). In honey bee *Apis mellifera* L. (Hymenoptera) exposed to 1000 µg AFB_1_/kg 30% mortality was observed (Fig. 2d), while exposure to 15,000 µg AFB_1_/kg caused 100% mortality after 60 h of treatment (Niu et al. 2011). Exposure to 1000 µg DON/kg did not affect survival in *Sitobion avenae* Fabr. nymphs, but caused 50% mortality in *Acyrthosiphon pisum* Harris nymphs. Mortality < 19% was observed for both species when exposed to 500–3000 µg/kg deoxynivalenol-3-glucoside (DON-3G) (De Zutter et al. 2016).

### Accumulation and metabolism

#### Diptera

**Fig. 3:**
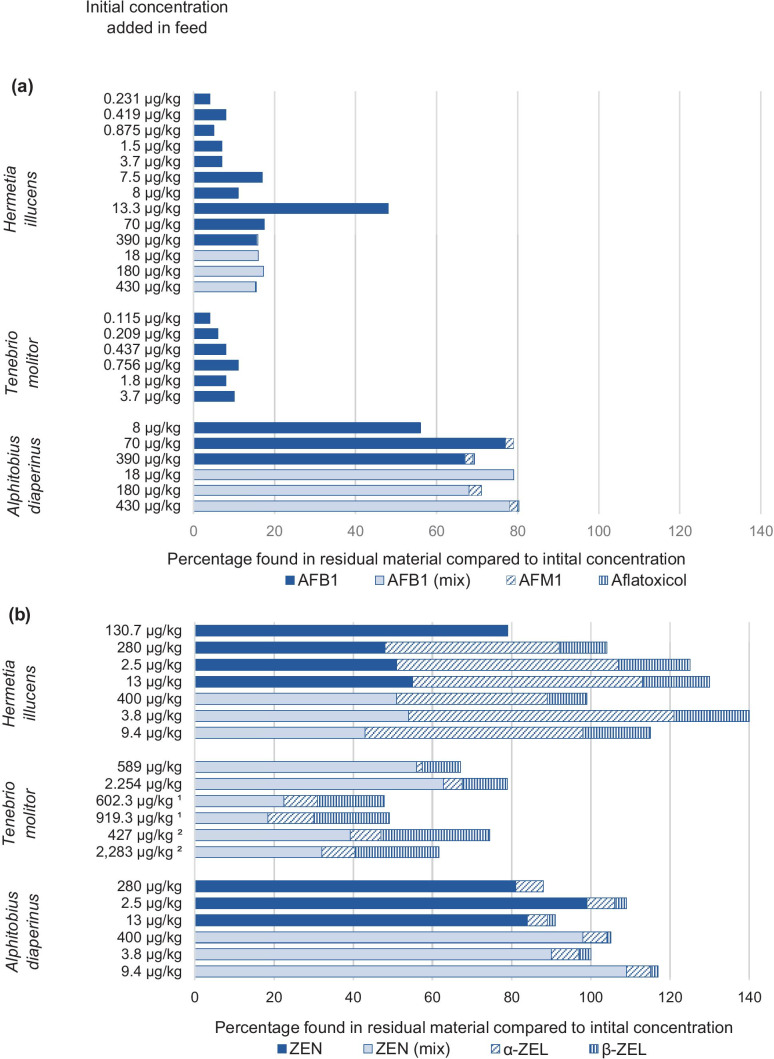
Overview of mycotoxin concentration in the feed substrate residues relative to the initial concentration (µg/kg) present in the feed substrate in percentages for *H. illucens*, *T. molitor*, and *A. diaperinus*. ^1^: naturally contaminated, ^2^: artificially contaminated, α-ZEL: α-zearalenol, β-ZEL: β-zearalenol; when not specified, the mycotoxins are spiked to the initial substrate. In none of the included studies, concentrations above the LOQ/LOD were found in the larvae; therefore, these percentages only represent the concentration found back in the residual material compared to the concentration in the initial substrate. In some of the studies, the final amount of residue was not mentioned. Based on the studies of **a**: Bosch et al. (2017), Camenzuli et al. (2018), Purschke et al. (2017); **b**: Camenzuli et al. (2018), Niermans et al. (2019), Purschke et al. (2017). An overview of the data used is available in Table S3 of the Supplementary Materials. Figure is made in Excel

In the order of Diptera, most studies on mycotoxin accumulation and metabolism were performed on *H. illucens* larvae. Concentrations observed were below the limit of quantification (LOQ) in *H. illucens* larval body when the larvae were given feed spiked with AFB_1_ (8–390 µg/kg), DON (3900–125,000 µg/kg), ZEN (280–13,000 µg/kg), or OTA (170–1300 µg/kg) either as single mycotoxin or in mixtures with different concentrations of AFB_1_, DON, ZEN, and OTA (Camenzuli et al. 2018). For the AFB1 treatments (Fig. 3a), only ≤ 18% of the initial concentration of AFB1 present in the substrate was found in residual feed material and neither aflatoxicol (AFL), aflatoxin M_1_ (AFM_1_) nor aflatoxin P_1_ (AFP_1_) was detected in levels above the LOQ in the residual material (Camenzuli et al. 2018). When *H. illucens* larvae were fed a naturally contaminated diet containing AFB_1_ (13.3 µg/kg), AFB_2_ (2.6 µg/kg), and AFG_2_ (7 µg/kg), 10.9 µg/kg AFB_1_ and concentrations below the LOQ for AFB_2_ and AFG_2_ were detected in the residual material (Purschke et al. 2017). Exposure of *H. illucens* larvae to substrates containing AFB_1_ as part of a mixture of mycotoxins (AFB_1_, DON, ZEN, and OTA) in different concentrations resulted in a lower concentration found in the residual material compared to the initial concentration than when fed single mycotoxins. However, when fed with a mixed diet containing 430 µg AFB_1_/kg, 100,000 µg DON/kg, 9400 µg ZEN/kg, and 2000 µg OTA/kg, a small amount of AFL was also formed (Camenzuli et al. 2018). A higher percentage of the initial concentration in the substrate was found back in the residual material of *H. illucens* larvae when DON was added as part of a mixture containing also AFB_1_, ZEN, and OTA (Camenzuli et al. 2018). When *H. illucens* larvae were exposed to a substrate with an initial concentration of 170–1700 µg OTA/kg, 41–62% was found back in the residual feed substrate (Camenzuli et al. 2018).

Correspondingly, feed spiked with concentrations of AFB_1_, DON, OTA, and ZEN either within the same range or lower than Camenzuli et al. (2018) resulted in non-detectable levels in *H. illucens* larvae (Purschke et al. 2017) and levels below the detection limit (LOD, limit of detection) when fed with 415 µg/kg AFB_1_ (Bosch et al. 2017). In a study performed by Leni et al. (2019), concentrations below the LOD were observed in the larval body of *H. illucens* fed on naturally contaminated substrates containing 779 µg/kg DON, 573 µg/kg FB_1_, and 441 µg/kg FB_2_ (Leni et al. 2019). It must be noted that data obtained for *H. illucens* larvae might not representative for all species within the order, since one study observed a tenfold higher concentration of AFB_1_ in 2nd instar *Musca domestica* L*.* larvae after only 2 days of exposure to 20 µg/kg AFB_1_ (Nevins and Grant 1971).

#### Coleoptera

Several studies investigating mycotoxin accumulation for species in the order Coleoptera were found; nevertheless, they only focus on *T. molitor* and *A. diaperinus*.

In 1st instar *T. molitor* larvae fed with 13 µg/kg AFB_1_, concentrations in the larval body were below the LOD. However, AFB_1_ was detected at 1% and 10% of the EC legal limit (being 20 µg/kg, Directive 2002/32/EC) after being fed with 23 or 415 µg/kg AFB_1_, respectively (Bosch et al. 2017). As shown in Fig. 3a, the percentage of AFB_1_ found back in the residual feed material of *T. molitor* larvae was low and seems to be dependent on the initial AFB_1_ concentration in the feed. In the residual feed material of *T. molitor* larvae, formation of small amounts of AFM_1_ was found; however, it must be noted that other AFB_1_ metabolites were not quantified in this study (Bosch et al. 2017).

As described for AFB_1_, also levels of DON were below the LOD in the larval body of *T. molitor* when exposed to up to 12,000 µg/kg DON (Niermans et al. 2019; Ochoa Sanabria et al. 2019; Van Broekhoven et al. 2017). However, the concentration of DON found in the residual feed material varied between studies. Van Broekhoven et al. (2017) showed that the percentage of excreted DON was lower in the residual feed material of larvae fed a naturally contaminated diet (ca. 14%) as opposed the percentage found in the residual feed material of larvae fed a spiked diet (ca. 41%) (Van Broekhoven et al. 2017). A second study found only a minor difference between the percentage of excreted DON when fed a spiked (58%) or a naturally contaminated (46–52%) diet after 8 weeks of exposure (Niermans et al. 2019). The percentage of excreted DON in *T. molitor* varied between 6.2 and 16.2% and appeared to decrease when fed with increasing concentrations, and the metabolite 3-acetyldeoxynivalenol (3-AcDON) was detected in the residual feed material in concentrations which were similar for all diets fed (Ochoa Sanabria et al. 2019). Since 3-AcDON was only chemically analyzed in residual feed materials of *T. molitor*, its presence cannot be compared with other species. Another study included the DON derivatives DON-3G and 15-acetyldeoxynivalenol (15-AcDON) in the analyses, but did not find them in the residual material (Van Broekhoven et al. 2017).

Similar trends were observed in *T. molitor* larvae when exposed to ZEN (Table S3). Levels detected in the larval body of *T. molitor* were below the LOD/LOQ when exposed to concentrations up to 2283 µg/kg ZEN. The percentage of excreted ZEN was lower in the residual feed material of larvae fed a naturally contaminated diet (19–23%) as opposed to the percentage found in residues of larvae fed a spiked diet (56–63%). As shown in Fig. 3b, formation of α- and β-zearalenol in different concentrations was observed in all diets fed (Niermans et al. 2019).

The study of Piacenza et al. (2020) showed that no T-2, HT-2, T-2 triol, or T-2 tetraol were detected (in levels above the LOD) in the surviving larvae or in the dead larvae collected from the control diets. However, after examination of the dead larvae collected from the other diets, 44.2 μg/kg T-2 was found in the body of the larvae fed with the naturally contaminated diet (250 μg/kg total T-2 and HT-2) and 7.7 μg/kg T-2 in the larvae fed the naturally contaminated diet (100 μg/kg total T-2 and HT-2) and the artificially contaminated diets. Additionally, T-2 and HT-2 were found in the residual material of both the naturally and artificially contaminated diets (except for the controls). The percentage of excreted T-2 found in the residual materials was higher when fed a naturally contaminated diet (51.7–66.5%) as opposed to an artificially contaminated diet (36.5–55.1%). HT-2 was only observed in the residual feed material of the larvae fed the artificially contaminated diets; interestingly, it seemed that the concentration found was not affected by the initial dose (Piacenza et al. 2020). Currently, no data are available on the accumulation or reduction of OTA in *T. molitor* residues. Data on FB_1_ are only available for *T. molitor* larvae and showed that when fed with doses varying between 50,000 and 450,000 µg FB_1_/kg approximately, 40% of the initial concentration was found back in the residual feed material. Accumulation within the larval body was not discussed and metabolites were not included in this study (Abado-Becognee et al. 1998).

Corresponding to what was found in *T. molitor* larvae, concentrations lower than the LOD/LOQ were observed in *A. diaperinus* larvae fed with substrates containing 8–390 µg/kg AFB_1_, 3900–125,000 µg/kg DON, 280–13,000 µg/kg ZEN, or 170–1300 µg/kg OTA tested alone and after exposure to combined mycotoxin concentrations up to 100,000 µg DON/kg (Camenzuli et al. 2018), 727 µg/kg FB_1_, and 294 µg/kg FB_2_ (Leni et al. 2019).

The study of Camenzuli et al. (2018) showed that in the residues of *A. diaperinus* larvae, AFM_1_ seems to be the main metabolite and AFL is also formed at the highest concentration (390 µg AFB_1_/kg). The authors also included the metabolites AFP_1_ and aflatoxin Q_1_ in their study, but the concentrations found were below the LOQ (5 µg/kg). *A. diaperinus* larvae were exposed to substrates containing AFB_1_ as part of a mixture of mycotoxins (AFB_1_, DON, ZEN, and OTA) in different concentrations. When compared with single AFB_1_ exposure of *A. diaperinus* larvae, the concentration found in the residual material was lower as compared to the initial concentration as when fed single mycotoxins. Also, formation of the same metabolites was observed. The percentage of excreted DON was similar when fed as single compound and when fed as part of a mixture and ranged between 80 and 96%. Concentrations of the included DON metabolites (3-AcDON, 15-AcDON and DON-3G) were below their respective LOQs (Camenzuli et al. 2018).

In residual feed material of *A. diaperinus* larvae, the percentage of α-zearalenol formed is similar when fed with initial concentrations ranging from 280 to 13,000 µg ZEN/kg. β-zearalenol was not formed, when an initial concentration of 280 µg/kg ZEN was fed to the larvae, and seems to be formed only at higher initial concentrations in the feed (Fig. 3b). Exposure to a mycotoxin mixture, resulted in a similar reduction of the concentration found in the residual material compared to the initial concentration of ZEN and formation of α-zearalenol and β-zearalenol in *A. diaperinus* as when spiked with ZEN alone (Camenzuli et al. 2018). For *A. diaperinus* larvae fed an OTA containing substrate (initial concentration of 170–1700 µg OTA/kg), 97–115% was found back in the residual material. Comparable results were found when OTA was fed as part of a mycotoxin mixture (AFB_1_, DON, and ZEN) (Camenzuli et al. 2018).

#### Lepidoptera

Only one study investigated mycotoxin accumulation in species of the order Lepidoptera, including *Hel. zea* and *O. nubilalis* larvae, reared on a diet containing 5000 µg/kg ZEN. ZEN was not detected in 4-day-old *O. nubilalis* larvae, whereas 650 µg ZEN/kg was observed in *Hel. zea* larvae of the same age. After 7 days of feeding, ± 600 µg ZEN/kg was also detected in *O. nubilalis* larvae. However, over time, a constant decrease of ZEN was observed in the larval body of both species. Feed residues were not analyzed in this study (Bily et al. 2004).

#### Other orders

When *P. americana* were fed with 12,000 µg AFB_1_/kg detectable levels up to 2 µg AFB_1_/kg were found in 40% of the tested insects, residual feed materials were not analyzed in this study (Llewellyn et al. 1976).

### Enzymes responsible for insect mycotoxin biotransformation

An explanation for the unrecovered fraction of mycotoxins could be that mycotoxin biotransformation occurs in the insects; therefore, the following section contains an overview of all studies suggesting enzymes responsible for mycotoxin biotransformation in insects (Table 2). The authors are not aware of any studies covering possible responsible enzyme systems in Coleoptera.

**Table 2:** Overview of suggested systems involved in mycotoxin metabolism in different insect species

Species	Mycotoxin	Enzyme system involved in mycotoxin metabolism and resulting metabolite if reported	Reference
**Diptera** *Hermetia illucens*	AFB_1_	Cyt P450 (AFP_1_); Cytoplasmic reductase (AFL)^1^	Meijer er at. (2019)
*Drosophila melanogaster* (strain Oregon R(R))	AFB_1_	Cyt P450 (CYP6A2)^8, 9^, depended on co-expression with a NADPH-Cyt P450-oxidoreductase	Saner et al. (1996)
**Lepidoptera** *Helicoverpa zea*	AFB_1_	Cyt P450^3^	Zeng et al. (2006)
*Helicoverpa zea*	AFB_1_	Cyt P450 (CYP321A1; AFP_1_)^1,4, 5^	Niu et al. (2008)
*Helicoverpa armigera*	AFB_1_	Cyt P450 (CYP6AE19)^4, 7^	Elzaki et al. (2019)
*Amyelois transitella*	AFB_1_	Cyt P450 (AFB_2a_, AFM_1_); NADPH-dependent reductase (AFL)^5^	Lee and Campbell (2000)
*Trichoplusia ni*	AFB_1_	Cyt P450 ^4^	Zeng et al. (2013)
**Other** *Apis mellifera*	AFB_1_	Cyt P450^3^	Niu et al. (2011)
*Apis mellifera*	AFB_1_	Cyt P450 (three CYP6AS)^8^	Johnson et al. (2012)
*Acyrthosiphon pisum* *Sitobion avenae*	DON	Glucosyltransferase (DON-3G)^1^	De Zutter et al. (2016)

*Cyt P450* cytochrome P450s

Methods of measurements: ^1^LC-MS/MS, ^2^Spectrophotometric enzyme assay, ^3^bioassays, ^4^RT-PCR, ^5^HPLC, ^6^Enzyme assays, ^7^gene-silencing, ^8^northern blotting, ^9^southern blotting

#### Diptera

Few studies regarding the metabolization of mycotoxins are available for *H. illucens* and *D. melanogaster*. Meijer et al. (2019) used an *H. illucens* S9 fraction, in combination with the cytochrome (Cyt) P450-enzyme inhibitor piperonyl butoxide (PBO) and showed that Cyt P450s were responsible for the metabolic conversion of AFB_1_ into AFP_1_ and pointed to a role of a cytoplasmic reductase for conversion of AFB_1_ into AFL (Meijer et al. 2019). In addition, the Cyt P450-enzyme CYP6A2 originating from the Oregon-R(R) strain of the fruit fly, *D. melanogaster*, seemed to be at least partially responsible for bioactivation of AFB_1_ to a recombinagen in a *Saccharomyces cerevisiae* strain; however, this metabolic activity seemed to be dependent on co-expression with a human-derived NADPH-Cyt P450-oxidoreductase (Saner et al. 1996).

#### Lepidoptera

ABF_1_ metabolism was not observed in midgut enzyme isolates from *Hel. zea* larvae fed with a control diet. However, AFB_1_ metabolism was observed in midgut isolates from larvae grown on diets supplemented with either coumarin or xanthotoxin in which the relatively non-toxic AFP_1_ was the main metabolite identified. Additionally, AFP_1_ formation was completely inhibited by the addition of PBO, required NADPH, and therefore indicated the role of Cyt P450s and more specifically CYP321A1 (Niu et al. 2008). The role of Cyt P450s in bioactivation of AFB_1_ in *Hel. zea* was identified after performance of a series of bioassays in which AFB_1_ toxicity was assessed in the presence of PBO (inhibitor) and phenobarbital (inducer). Addition of PBO caused a significantly decreased toxicity and an increased pupation rate in 4th and 5th instar *Hel. zea* larvae (Zeng et al. 2006). Corresponding to what was mentioned previously, also a second study showed that PBO reduced the toxicity of AFB_1_ to 5-day-old *Tr. ni* larvae (Zeng et al. 2013). When *H. armigera* larvae were fed a diet containing AFB_1_ and injected with ds*CYP6AE19* to silence *CYP6AE19* expression, a decreased mortality was found compared to when fed the same diet but injected with ds*GFP* (green fluorescent protein) or water suggesting that induction of CYP6AE19 results in a higher toxicity of AFB_1_ (Elzaki et al. 2019). In an in vitro inhibition study of AFB_1_ metabolism, larvae of *Am. transitella* produced AFB_2a_, AFM_1_, and mostly AFL, while for *Cydia pomonella*, only AFL was detected at trace level. After adding PBO, production of AFB_2a_ and AFM_1_ by *Am. transitella* was completely inhibited, indicating a role for Cyt P450s. Additionally, the role of NAD(P)H and glutathione (GSH) in AFL production was tested and showed that NADPH and GSH were equally effective in AFL production in *Am. transitella*, whereas a mixture of both enhanced AFL production. The authors concluded that a NADPH-dependent reductase seems to be responsible for the transformation of AFB_1_ into AFL and suggests the involvement of GSH as an electron donor in AFL formation (Lee and Campbell 2000).

#### Other orders

The role of a phase II enzyme glucosyltransferase was proposed to be involved in the detoxification of DON into DON-3G in the aphids *Si. avenae* and *Ac. pisum.* Interestingly, *Si. avenae*, which co-occurs with the DON-producing *Fusarium graminearum*, converted DON to DON-3G more efficiently than *Ac. pisum* which normally feeds on plants not considered as host for *F. graminearum* (De Zutter et al. 2016). It was hypothesized that natural phytochemicals which are present in the insects’ food could possibly induce Cyt P450 activity and help in the detoxification of AFB_1_. To support this statement, the effect of AFB_1_ in combination with honey was examined and it was observed that *A. mellifera* adults fed on honey were more tolerant to AFB_1_ exposure than bees fed on other diets. An elevated expression of three *CYP6AS* P450 genes were observed in northern blot analyses of the guts of bees fed extracts of honey, pollen, and propolis and suggested that consumption of possible phytochemicals present in honey can induce Cyt P450s responsible for detoxification of AFB_1_ in *A. mellifera* (Johnson et al. 2012). In addition, a role for Cyt P450s was indicated in AFB_1_ detoxification in *A. mellifera*. A decreased survival time was observed in *A. mellifera* after consumption of bee candy containing 10,000 μg/kg AFB_1_ supplemented with either 0.05% PBO or 0.1% PBO as compared to when bee candy with the same concentration of AFB_1_ was consumed alone. Additionally, the authors fed *A. mellifera* with two concentrations of OTA (10,000 and 40,000 μg/kg) supplemented with either 0.05% PBO or 0.1% PBO, but in this case, the addition of PBO did not seem to affect survival time (Niu et al. 2011).

## Discussion

### Tolerance

The insect sector offers potential to promote circular and sustainable opportunities for feed production. Also, the contribution to organic waste management is interesting from an economic and ecological point of view. When using waste or side streams as a substrate for insect rearing low-quality streams can be upgraded into high-quality protein or fat fractions. Another ecological advance supported by this review is that insects can breakdown complex mycotoxins and metabolize them into smaller less- or non-toxic metabolites. However, it needs to be clarified whether insects fed on these possibly contaminated waste or side streams show a high tolerance and yield enough biomass for economically feasible production.

This systematic review summarized published data about tolerance to mycotoxins for insect species mainly belonging to three insect orders. Although the available information is rather limited, tolerance differences between orders, within orders, and even between strains and stages of the same species become apparent (Fig. 2a). In addition to differences between insect species, growth and mortality were affected differently by the type of mycotoxin, the concentration of the mycotoxin fed, and the life-stage in which mycotoxin exposure occurred. The studies on Coleoptera showed a reduction as well as an increase in biomass after exposure to mycotoxin contaminated diets. The actual effect depended on the mycotoxin and its concentration and whether the toxin was present naturally or spiked to the substrate. Growth effects caused by mycotoxin exposure of Lepidoptera were reported in several studies and showed clear variation in tolerance between species, larval stages, and mycotoxins. Overall, insects of the order Coleoptera show lower mortality after exposure to AFB_1_ when compared to Lepidoptera and Diptera. Additionally, the inclusion of certain supplements in the mycotoxin-contaminated diet showed to have an influence as exposure of *Tr. ni* larvae to a diet containing the plant allelochemical xanthotoxin in combination with AFB_1_ resulted in a substantially higher weight and pupation rate when compared to AFB_1_ exposure alone (Zeng et al. 2013).

All papers which discussed the effect of mycotoxin exposure on insects are included in this review. In some papers, the application of mycotoxins for biocontrol of insects is the main focus. These papers included mycotoxins such as roseotoxin B and brevianamides which are currently not considered as food-relevant. However, the future aim is to use (contaminated) organic waste and/or side streams as substrate for insect rearing, rather than substrates from a solely food-relevant origin. Some of these mycotoxins are relevant for biocontrol of insects and therefore might affect insect tolerance and/or growth. As discussed in this review, exposure of *S. littoralis* larvae to 10,000 µg/kg penicillic acid or brevianamide A led to a mortality of 90% and 78% respectively (Paterson et al. 1987), which suggests that substrates containing these toxins in a similar or higher concentration might be unsuitable for insect rearing. Overall, the data discussed provides a positive outlook for the use of mycotoxin contaminated organic waste and/or side streams in the future.

### Type of substrate

Substrate materials investigated in the retrieved studies ranged from poorly defined waste streams to refined sugar. The type of substrate fed could have had an effect on the study outcome, as was shown in the case where mortality of *A. mellifera* exposed to AFB_1_ in honey was lower than when fed with AFB_1_ in sucrose (Johnson et al. 2012). In the retrieved studies, insects were exposed via artificially contaminated, spiked and naturally contaminated substrates, resulting in heterogenous effects. *T. molitor* larvae reared on wheat naturally contaminated with mycotoxins gained significantly more weight than when reared on spiked or artificially contaminated wheat (Niermans et al. 2019; Van Broekhoven et al. 2017). Naturally contaminated substrates might contain a mixture of mycotoxins or modified forms that could have had a synergistic effect on the larvae when exposed to them, which offers the insect a very different situation as compared to being fed a substrate spiked with a single mycotoxin. Accordingly, the studies included in this review showed that the presence of multiple mycotoxins in the insect diet, compared to the presence of a single mycotoxin, influenced mortality as was shown when *Hel. zea* larvae fed on a substrate contaminated with fusaric acid and the plant secondary metabolite gossypol experienced 18% mortality as opposed to fusaric acid or gossypol alone not leading to mortality (Dowd 1988). The results obtained from feeding studies in which the substrate was spiked with a single mycotoxin may therefore be not fully representative for when waste or side streams are used. However, using spiked feed is more controlled and is necessary for a first exploration of the effects of single mycotoxins on insect tolerance and metabolism.

### Accumulation/biotransformation

When insects are sold for human or animal consumption, they need to comply to the MLs and guidance levels set in the respective legislations, which makes no/low mycotoxin accumulation an important requirement. Mycotoxin accumulation in the insect body was observed in some specific cases; however, the concentration in the insects found was mostly below their respective MLs or guidance values. In general, the available data demonstrate that mycotoxin levels in the insect larvae are below the respective LOD/LOQ, even when exposed to concentrations above the European Commission ML for the presence of mycotoxins in food and their commodities, and guidance values set for mycotoxins in feed (EC 2006b; EU 2002). Additionally, the available studies have shown that only a fraction of the initially added mycotoxin concentration in the feed was found back in the residual feed material (Fig. 3a), even when taking into account main metabolites that could be formed. The unrecovered fraction could be explained in multiple ways; interference by the matrix leading to a loss of signal, breakdown of parent compounds, transformation into modified forms and/or the formation of unknown metabolites, amongst others. The unrecovered fraction was larger for DON, OTA, and AFB_1_ in *H. illucens* and *T. molitor* larvae as compared to *A. diaperinus* larvae. Formation of the toxic metabolite α-zearalenol was highest in the residual feed material of *H. illucens* when compared to the concentrations found in the residues of *T. molitor* and *A. diaperinus* (Fig. 3b) (Camenzuli et al. 2018). Identification and quantification of unknown metabolites will allow for a more complete mass balance in the future and will give a better insight in the possible detoxification by insects.

### Enzyme systems

Insects have developed metabolic adaptations that can result in detoxification and/or yield metabolites that are easier to transport or excrete (Birnbaum and Abbot 2018). Metabolites formed can be more or less toxic than the parent compound (in this case the original mycotoxin in the substrate), and they should be identified and investigated for toxicity to ensure safety when insects are used as food or feed ingredients. Identification of enzyme systems responsible for the formation of mycotoxin metabolites will foster insights in the pathways involved in mycotoxin metabolism in the insect body and, hence, the possible metabolites that are formed. To date, most studies that identified enzyme systems involved in mycotoxin metabolism mainly focused on AFB_1_ (Table 2). In insects, enzymes for phase I metabolism, Cyt P450s, as well as phase II enzymes, glycosyltransferases, are known to transform AFB_1_. A recently published review covering Cyt P450-mediated mycotoxin metabolism in plant-feeding insects concluded that the involved Cyt P450s mostly belong to families known to detoxify phytochemicals (Berenbaum et al. 2021), which is in accordance with data found in this review. Cyt P450s were involved in the conversion of AFB_1_ to mostly AFP_1_ and the roles of GSH and NADH were identified in the formation of AFL in *Am. transitella*. Cyt P450 enzymes and their subfamilies are also found in most tissues of various animal species where they play a role in mycotoxin metabolism (Hussein and Brasel 2001). As an example, chicken and quail hepatic microsomes use CYP2A6 and to a lesser extent CYP1A to transform AFB_1_ into the extremely reactive AFB_1_-8–9-epoxide (Diaz et al. 2010). A cytosolic reductase important in the reduction of AFB_1_ to AFL seems to be produced in poultry as well, however, in larger quantities in turkey and duck then in quail and chicken (Peles et al. 2019). In bovine hepatocytes, AFM_1_, mainly formed by CYP1A and CYP3A hepatic monooxygenase activities, seemed to be the most prominent metabolite formed within the first hours of incubation (Kuilman et al. 2000). Additional to the shared importance of the Cyt P450 enzymes, cytoplasmic reductases, and GSH, no solid conclusions can as yet be made on the comparison between the metabolism of mycotoxins by insects and another animals.

### Other uncertainties

The LOD of the analytical system used to determine mycotoxin concentrations in the substrates, larval material, and the residues is a critical point in this discussion. Although Table S1 gives an extensive overview of all analytical methods used, the relevant LODs are not always known. A weak analysis can result in a no-toxin level in the larvae, while in reality, the method or machine used might have a limited sensitivity. A sensitive method of analysis is especially important for measuring the presence of aflatoxins since very low concentrations are already unwanted when insects are used for used for food and/or feed purposes later on (Table 1). However, most recent studies do provide information on the sensitivity and detection limits of the analyses performed and are able to detect mycotoxin concentrations in insects in levels far below their respective legal limits (Camenzuli et al. 2018; Meijer et al. 2019). Finally, the question remains whether the entire amount of feed (and therefore the present toxin) was consumed by the insects during the exposure period and how this, when not fully consumed, would affect the data obtained in the discussed studies.

### Outlook

This study presents comprehensive data on the effects of mycotoxins on insect growth and survival, as well as mycotoxin accumulation and conversion by insects. Most data relate to species which are agricultural pests and species potentially used as food or feed in the EU. Survival and growth as well as tolerance and metabolization vary between species, between mycotoxins, and their concentration as well as the type of substrate used, whether the mycotoxin was present naturally or spiked, and the presence of possible supplements. Accumulation of mycotoxins was identified as mostly below LOD/LOQ for the included species. Since data cannot be generalized across species and not even across strains of the same species, additional studies on other insect species than the main species covered in this review (*H. illucens*, *T. molitor*, and *A. diaperinus*) are recommended, specifically on insects possibly considered for food/feed including crickets and locusts. Cytochrome P450s were suggested as main enzymes involved in AFB_1_ metabolism in some insects; however, further research is recommended on unravelling metabolic pathways, involvement of phase II enzymes, the formation of possible unknown metabolites, and their toxicity. Overall, based on the available data, the use of mycotoxin contaminated waste streams as substrate for insect rearing seems to provide a promising approach for the future of mycotoxin remediation and a circular economy.

## Supplementary Information

Below is the link to the electronic supplementary material.

Supplementary file1 (XLSX 738 KB)

Supplementary file2 Table S1: Overview of covered studies including: insect species, substrate used, exposure time of the insects, analytical method used, and mycotoxins (metabolites) analyzed. (PDF 195 KB)

Supplementary file3 (PDF 205 KB)

Supplementary file4 Table S2: Overview of data on mortality after AFB1 exposure for insects in the orders Diptera, Coleoptera, Lepidoptera and species Apis mellifera. (PDF 184 KB)

Supplementary file5 Table S3: Concentrations of parent compounds and metabolites in initial substrate, larvae, and residual material with mass balance provided of Hermetia illucens, Tenebrio molitor, and Alphitobius diaperinus. (PDF 341 KB)

## References

[CR1] Abado-Becognee K, Fleurat-Lessard F, Creppy EE, Melcion D (1998). Effects of fumonisin B_1_ on growth and metabolism of larvae of the yellow mealworm, Tenebrio molitor. Entomol Exp Appl.

[CR2] Agriopoulou S, Stamatelopoulou E, Varzakas T (2020). Advances in occurrence, importance, and mycotoxin control strategies: prevention and detoxification in foods. Foods.

[CR3] Berenbaum MR, Bush DS, Liao L-H (2021). Cytochrome P450-mediated mycotoxin metabolism by plant-feeding insects. Curr Opin Insect Sci.

[CR4] Bily AC (2004). Analysis of Fusarium graminearum mycotoxins in different biological matrices by LC/MS. Mycopathologia.

[CR5] Birnbaum SSL, Abbot P (2018). Insect adaptations toward plant toxins in milkweed–herbivores systems – a review. Entomol Exp Appl.

[CR6] Bosch G, Fels-Klerx HJV, Rijk TC, Oonincx D (2017). Aflatoxin B_1_ tolerance and accumulation in black soldier fly larvae (Hermetia illucens) and yellow mealworms (Tenebrio molitor). Toxins (basel).

[CR7] Camenzuli L, van Dam R, de Rijk T, Andriessen R, van Schelt J, van der Fels-Klerx HJI (2018). Tolerance and excretion of the mycotoxins aflatoxin B_1_, zearalenone, deoxynivalenol, and ochratoxin A by *Alphitobius diaperinus* and *Hermetia illucens* from contaminated substrates. Toxins (basel).

[CR8] Cao LJ, Jiang W, Hoffmann AA (2019). Life history effects linked to an advantage for wAu Wolbachia in Drosophila. Insects.

[CR9] Chinnici JP, Erlanger L, Charnock M, Jones M, Stein J (1979). Sensitivity differences displayed by Drosophila melanogaster larvae of different ages to the toxic effects of growth on media containing aflatoxin B_1_. Chem Biol Interact.

[CR10] Cito A, Barzanti GP, Strangi A, Francardi V, Zanfini A, Dreassi E (2016). Cuticle-degrading proteases and toxins as virulence markers of Beauveria bassiana (Balsamo) Vuillemin. J Basic Microbiol.

[CR11] Davis GRF, Schiefer HB (1982). Effects of dietary T-2 toxin concentrations fed to larvae of the yellow mealworm at three dietary protein levels. Comp Biochem Physiol C Toxicol.

[CR12] De Zutter N, Audenaert K, Arroyo-Manzanares N, De Boevre M, Van Poucke C, De Saeger S, Haesaert G, Smagghe G (2016). Aphids transform and detoxify the mycotoxin deoxynivalenol via a type II biotransformation mechanism yet unknown in animals. Sci Rep.

[CR13] Diaz GJ, Murcia HW, Cepeda SM (2010). Cytochrome P450 enzymes involved in the metabolism of aflatoxin B_1_ in chickens and quail. Poult Sci.

[CR14] Dowd PF (1988). Toxicological and biochemical interactions of the fungal metabolites fusaric acid and kojic acid with xenobiotics in Heliothis zea (F.) and Spodoptera frugiperda (J.E. Smith). Pestic Biochem Physiol.

[CR15] Dowd PF (1989). Fusaric acid - a secondary fungal metabolite that synergizes toxicity of cooccurring host allelochemicals to the corn earworm, Heliothis zea (Lepidoptera). J Chem Ecol.

[CR16] Dowd PF (1990). Responses of representative midgut detoxifying enzymes from Heliothis zea and Spodoptera frugiperda to trichothecenes. Insect Biochem.

[CR17] Dowd PF (1993). Toxicity of the fungal metabolite griseofulvin to Helicoverpa zea and Spodoptera frugiperda. Entomol Exp Appl.

[CR18] Dowd PF, Cole RJ, Vesonder RF (1988). Toxicity of selected tremorgenic mycotoxins and related compounds to Spodoptera frugiperda and Heliothis zea. J Antibiot (tokyo).

[CR19] EC – European Commission (2006a) Commission recommendation 2006/576/EC of 17 August 2006 on the presence of deoxynivalenol, zearalenone, ochratoxin A, T-2 and HT-2 and fumonisins in products intended for animal feeding. Off J Eur Union L 229/7. Last consolidated version available from: https://eur-lex.europa.eu/legal-content/EN/TXT/PDF/?uri=CELEX:32006H0576&from=EN

[CR20] EC – European Commission (2006b) Commission Regulation (EC) No 1881/2006 of 19 December 2006 setting maximum levels for certain contaminants in foodstuffs. Off J Eur Union L 364/5. Last consolidated version available from: https://eur-lex.europa.eu/legal-content/EN/TXT/PDF/?uri=CELEX:02006R1881-20201014&from=EN

[CR21] EFSA – European Food Safety Authority, Panel on Contaminants in the Food Chain (2014). Scientific opinion on the risks to human and animal health related to the presence of beauvericin and enniatins in food and feed. EFSA J.

[CR22] EFSA – European Food Safety Authority, Panel on Contaminants in the Food Chain (2016). Scientific opinion on the appropriateness to set a group health-based guidance value for zearalenone and its modified forms. EFSA J.

[CR23] Elzaki MEA, Xue RR, Hu L, Wang JD, Zeng RS, Song YY (2019). Bioactivation of aflatoxin B_1_ by a cytochrome P450, CYP6AE19 induced by plant signaling methyl jasmonate in Helicoverpa armigra (Hübner). Pestic Biochem Physiol.

[CR24] EU - The European Parliament and the Council of the European Union (2002). Directive 2002/32/EC of the European Parliament and of the Council of 7 May 2002 on undesirable substances in animal feed. Off J Eur Union L 140. Last consolidated version available from: https://eur-lex.europa.eu/legal-content/EN/TXT/PDF/?uri=CELEX:02002L0032-20191128&from=EN

[CR25] Gruber-Dorninger C, Jenkins T, Schatzmayr G (2019). Global mycotoxin occurrence in feed: a ten-year survey. Toxins (basel).

[CR26] Gulsunoglu Z, Aravind S, Bai Y, Wang L, Kutcher HR, Tanaka T (2019). Deoxynivalenol (DON) accumulation and nutrient recovery in black soldier fly larvae (Hermetia illucens) fed wheat infected with Fusarium spp. Fermentation.

[CR27] Gunst K, Chinnici JP, Llewellyn GC (1982). Effects of aflatoxin B_1_, aflatoxin B_2_, aflatoxin G_1_, and sterigmatocystin on viability, rates of development, and body length in two strains of Drosophila melanogaster (Diptera). J Invertebr Pathol.

[CR28] Hegde UC, Chandra T, Shanmugasundaram ER (1967). Toxicity of different diets contaminated with various fungi to rice moth larvae (Corcyra cephalonica st). Can J Comp Med Vet Sci.

[CR29] Hussein HS, Brasel JM (2001). Toxicity, metabolism, and impact of mycotoxins on humans and animals. Toxicology.

[CR30] Janković-Tomanić M, Petković B, Todorović D, Vranković J, Perić-Mataruga V (2019). Physiological and behavioral effects of the mycotoxin deoxynivalenol in Tenebrio molitor larvae. J Stored Prod Res.

[CR31] Johnson RM, Mao W, Pollock HS, Niu G, Schuler MA, Berenbaum MR (2012) Ecologically appropriate 943 xenobiotics induce cytochrome P450s in Apis mellifera. PLoS One 7:e31051. 10.1371/journal.pone.0031051 10.1371/journal.pone.0031051 PMC3272026 22319603

[CR32] Jongema Y (2017) List of edible insect species of the world. Laboratory of Entomology, Wageningen UR, Wageningen, the Netherlands. Available via: https://onlinelibrary.wiley.com/doi/10.1002/1520-6327(200012)45:4%3C166::AIDARCH4%3E3.0.CO;2-8

[CR33] Kanaoka M, Isogai A, Murakoshi S, Ichinoe M, Suzuki A, Tamura S (1978). Bassianolide, a new insecticidal cyclodepsipeptide from Beauveria bassiana and Verticillium lecanii. Agr Biol Chem.

[CR34] Kauppi SM, Pettersen I, Boks C (2019). Consumer acceptance of edible insects and design interventions as adoption strategy. Int J Food Des.

[CR35] Kirk HD, Ewen AB, Emson HE, Blair DGR (1971). Effect of aflatoxin B_1_ on development of Drosophila melanogaster (Diptera). J Invertebr Pathol.

[CR36] Kuilman MEM, Maas RFM, Fink-Gremmels J (2000). Cytochrome P450-mediated metabolism and cytotoxicity of aflatoxin B_1_ in bovine hepatocytes. Toxicol in Vitro.

[CR37] Lee SU, Campbell BC (2000) In vitro metabolism of aflatoxin B_1_ by larvae of navel orangeworm, *Amyelois transitella* (Walker) (Insecta, Lepidoptera, Pyralidae) and codling moth, Cydia pomonella (L.) (Insecta, Lepidoptera, Tortricidae). Arch Insect Biochem Physiol 45:166–174. https://onlinelibrary.wiley.com/doi/10.1002/1520-6327(200012)45:4%3C166::AIDARCH4%3E3.0.CO;2-8 10.1002/1520-6327(200012)45:4<166::AID-ARCH4>3.0.CO;2-8 11223936

[CR38] Leni G, Cirlini M, Jacobs J, Depraetere S, Gianotten N, Sforza S, Dall’Asta C, (2019). Impact of naturally contaminated substrates on Alphitobius diaperinus and Hermetia illucens: uptake and excretion of mycotoxins. Toxins (basel).

[CR39] Llewellyn GC, Gee CL, Sherertz PC (1988). Toxic responses of developing fifth instar milkweed bugs, Oncopeltus fasciatus (Hemiptera), to aflatoxin B_1_. Bull Environ Contam Toxicol.

[CR40] Llewellyn GC, Sherertz PC, Mills RR (1976). The response of dietary stressed Periplaneta americana to chronic intake of pure aflatoxin B_1_. Bull Environ Contam Toxicol.

[CR41] Madau FA, Arru B, Furesi R, Pulina P (2020). Insect farming for feed and food production from a circular business model perspective. Sustainability.

[CR42] Matsumura F, Knight SG (1967). Toxicity and chemosterilizing activity of aflatoxin against insects. J Econ Entomol.

[CR43] Medina A, Akbar A, Baazeem A, Rodríguez A, Magan N (2017) Climate change, food security and mycotoxins: do we know enough? Fungal Biol Rev 31:143–154. 10.1016/j.fbr.2017.04.002

[CR44] Meijer N, Stoopen G, van der Fels-Klerx HJ, van Loon JJA, Carney J, Bosch G (2019). Aflatoxin B_1_ conversion by black soldier fly (Hermetia illucens) larval enzyme extracts. Toxins (basel).

[CR45] Melone PD, Chinnici JP (1986). Selection for increased resistance to aflatoxin B_1_ toxicity in Drosophila melanogaster. J Invertebr Pathol.

[CR46] Mencarelli M, Accinelli C, Vicari A (2013). Implications of European corn borer, Ostrinia nubilalis, infestation in an Aspergillus flavus-biocontrolled corn agroecosystem. Pest Manag Sci.

[CR47] Miller JD, Sumarah MW, Adams GW (2008). Effect of a rugulosin-producing endophyte in Picea glauca on Choristoneura fumiferana. J Chem Ecol.

[CR48] Nevins MP, Grant DW (1971). Bioconcentration and biotransfer of aflatoxin. Bull Environ Contam Toxicol.

[CR49] Niermans K, Woyzichovski J, Kröncke N, Benning R, Maul R (2019). Feeding study for the mycotoxin zearalenone in yellow mealworm (Tenebrio molitor) larvae—investigation of biological impact and metabolic conversion. Mycotoxin Res.

[CR50] Niu G, Johnson RM, Berenbaum MR (2011). Toxicity of mycotoxins to honeybees and its amelioration by propolis. Apidologie.

[CR51] Niu G, Siegel J, Schuler MA, Berenbaum MR (2009). Comparative toxicity of mycotoxins to navel orangeworm (Amyelois transitella) and corn earworm (Helicoverpa zea). J Chem Ecol.

[CR52] Niu G, Wen Z, Rupasinghe SG, Ren SZ, Berenbaum MR, Schuler MA (2008). Aflatoxin B_1_ detoxification by CYP321A1 in Helicoverpa zea. Arch Insect Biochem Physiol.

[CR53] Niyonsaba HH, Höhler J, Kooistra J, Van der Fels-Klerx HJ, Meuwissen MPM (2021). Profitability of insect farms. J Insects as Food Feed.

[CR54] Ochoa Sanabria C, Hogan N, Madder K, Gillott C, Blakley B, Reaney M, Beattie A, Buchanan F (2019). Yellow mealworm larvae (Tenebrio molitor) fed mycotoxin-contaminated wheat-a possible safe, sustainable protein source for animal feed?. Toxins (basel).

[CR55] Ohtomo T, Murakoshi S, Sugiyama J, Kurata H (1975). Detection of aflatoxin B_1_ in silkworm larvae attacked by an Aspergillus flavus isolate from a sericultural farm. Appl Microbiol.

[CR56] Paterson RRM, Simmonds MJS, Kemmelmeier C, Blaney WM (1990). Effects of brevianamide A, its photolysis product brevianamide D, and ochratoxin A from two Penicillium strains on the insect pests Spodoptera frugiperda and Heliothis virescens. Mycol Res.

[CR57] Paterson RRM, Simmonds MSJ, Blaney WM (1987). Mycopesticidal effects of characterized extracts of Penicillium isolates and purified secondary metabolites (including mycotoxins) on Drosophila melanogaster and Spodoptora littoralis. J Invertebr Pathol.

[CR58] Peles F, Sipos P, Győri Z, Pflieger WP, Giacometti F, Serraino A, Pagliuca G, Gazzotti T, Pócsi I (2019). Adverse effects, transformation and channeling of aflatoxins into food raw materials in livestock. Front Microbiol.

[CR59] Piacenza N, Kaltner F, Maul R, Gareis M, Schwaiger K, Gottschalk C (2020). Distribution of T-2 toxin and HT-2 toxin during experimental feeding of yellow mealworm (Tenebrio molitor). Mycotoxin Res.

[CR60] Purschke B, Scheibelberger R, Axmann S, Adler A, Jäger H (2017). Impact of substrate contamination with mycotoxins, heavy metals and pesticides on the growth performance and composition of black soldier fly larvae (Hermetia illucens) for use in the feed and food value chain. Food Addit Contam Part A Chem Anal Control Expo Risk Assess.

[CR61] Rizwan-Ul-Haq M, Hu QB, Hu MY, Zhong G, Weng Q (2009). Study of destruxin B and tea saponin, their interaction and synergism activities with Bacillus thuringiensis kurstaki against Spodoptera exigua (Hübner) (Lepidoptera: Noctuidae). Appl Entomol Zool.

[CR62] Sadek MM (1996). The chemosterilizing activity of some mycotoxins and their influence on the development and survival of Spodoptera littoralis (Boisd.) (Lep., Noctuidae). J Appl Entomol.

[CR63] Saner C, Weibel B, Würgler FE, Sengstag C (1996) Metabolism of promutagens catalyzed by Drosophila melanogaster CYP6A2 enzyme in Saccharomyces cerevisiae. Environ Mol Mutagen 27:46–58. 10.1002/(SICI)1098-2280(1996)27:1<46::AID-EM7>3.0.CO;2-C 10.1002/(SICI)1098-2280(1996)27:1<46::AID-EM7>3.0.CO;2-C 8625948

[CR64] Schrögel P, Wätjen W (2019). Insects for food and feed-safety aspects related to mycotoxins and metals. Foods.

[CR65] Şişman T (2006). The protective effect of hydrated sodium calcium aluminosilicate against the adverse effects of aflatoxin B_1_ on D. melanogaster. Toxicol Ind Health.

[CR66] Sree KS, Padmaja V (2008). Destruxin from Metarhizium anisopliae induces oxidative stress effecting larval mortality of the polyphagous pest Spodoptera litura. J Appl Entomol.

[CR67] Sumarah M, Adams G, Berghout J, Slack G, Wilson A, Miller J (2008). Spread and persistence of a rugulosin-producing endophyte in Picea glauca seedlings. Mycol Res.

[CR68] Van Broekhoven S, Doan QH, van Huis A, van Loon JJ (2014) Exposure of tenebrionid beetle larvae to mycotoxin-contaminated diets and methods to reduce toxin levels. Neth Entomol Soc Meet 25:47-58

[CR69] Van Broekhoven S, Mota Gutierrez J, De Rijk TC, De Nijs WCM, Van Loon JJA (2017). Degradation and excretion of the Fusarium toxin deoxynivalenol by an edible insect, the yellow mealworm (Tenebrio molitor L.). World Mycotoxin J.

[CR70] van der Fels-Klerx HJ, Camenzuli L, Belluco S, Meijer N, Ricci A (2018). Food safety issues related to uses of insects for feeds and foods. CRFSFS.

[CR71] Van Huis A (2016). Edible insects are the future?. Proc Nutr Soc.

[CR72] Van Huis A (2020). Insects as food and feed, a new emerging agricultural sector: a review. J Insects as Food Feed.

[CR73] Van Huis A, Oonincx DGAB (2017). The environmental sustainability of insects as food and feed. A Review Agron Sustain Deve.

[CR74] Van Huis A, Van Itterbeeck J, Klunder H, Mertens E, Halloran A, Muir G, Vantomme P (2013) Edible insects: future prospects for food and feed security. (FAO forestry paper; No. 171). Food and Agriculture Organization of the United Nations. https://edepot.wur.nl/258042

[CR75] Wright VF, De Las CE, Harein PK (1976). The response of Tribolium confusum to the mycotoxins zearalenone (F-2) and T-2 toxin. Environ Entomol.

[CR76] Zeng RS, Niu G, Wen Z, Schuler MA, Berenbaum MR (2006). Toxicity of aflatoxin B_1_ to Helicoverpa zea and bioactivation by cytochrome P450 monooxygenases. J Chem Ecol.

[CR77] Zeng RS, Wen Z, Niu G, Berenbaum MR (2013). Aflatoxin B_1_: toxicity, bioactivation and detoxification in the polyphagous caterpillar Trichoplusia ni. Insect Sci.

[CR78] Zeng RS, Wen Z, Niu G, Schuler MA, Berenbaum MR (2009). Enhanced toxicity and induction of cytochrome P450s suggest a cost of “eavesdropping” in a multitrophic interaction. J Chem Ecol.

[CR79] Zhao X, Wang D, Fields PG, Li H (2018). Effect of aflatoxin B_1_ on development, survival and fecundity of Ahasverus advena (Waltl). J Stored Prod Res.

