# Supplementary material for: A systematic literature review on the effects of mycotoxin exposure on insects and on mycotoxin accumulation and biotransformation
Source: Mycotoxin Res. 2021 Oct 7;37(4):279–95. doi: 10.1007/s12550-021-00441-z (PMC8571154; doi:10.1007/s12550-021-00441-z)
Supplement: Supplementary file 3 — Supplementary file3 (PDF 205 KB) [file 12550_2021_441_MOESM3_ESM.pdf]

Mycotoxin Research

A Systematic Literature Review on the Effects of Mycotoxin Exposure on Insects, and on Mycotoxin Accumulation and Biotransformation

K. Niermans[1,2], A.M. Meyer[2], E.F. Hoek- van den Hil[2], J.J.A. van Loon[1], H.J. van der Fels-Klerx[2]

[1] Wageningen University, Department of Plant Sciences, Laboratory of Entomology, Wageningen, The Netherlands

[2] Wageningen Food Safety Research, Akkermaalsbos 2, 6708 WB, Wageningen, The Netherlands

Corresponding Author: ine.vanderfels@wur.nl

Supplementary Table S1: Overview of covered studies including: insect species, substrate used, exposure time of the insects, analytical method used, and mycotoxins (metabolites) analyzed

Reference	Orders	Species	Substrates	Insect stage	Time of exposure	Method of measurement	Mycotoxins Analyzed [1]	Mycotoxin concentrations in feed	Fungus present
Abado-Becognee et al. (1998)	Coleoptera	*Tenebrio molitor*	Maize flour with 4% (w/w) mineral salt and 13% vitamin B complex	Larvae (7 weeks)	63 days	HPLC w Fluorescence Detector	FB_1	FB_1: 50,000, 150,000, 450,000 µg/kg	No
Bily et al. (2004)	Lepidoptera	*Helicoverpa zea* and *Ostrinia nubilalis*	*Hel. zea*: soybean flour/wheat germ artificial diet.	Larvae (3rd instar)	4, 7, 11 and 14 days	LC-MS ~~w APCI mass detector~~	ZEN	ZEN: 5,000 µg/kg	No

			O. nubilalis: Meredic diet						
Bosch et al. (2017)	Coleoptera, Diptera	*Tenebrio molitor* and *Hermetia illucens*	Poultry feed	Larvae (1st instar)	*T. molitor*: 40 days; *H. illucens*: 10 days	HPLC w Fluorescence Detector	AFB_1, AFM_1	AFB_1: 10, 25, 50, 100, 250 and 500 ug/kg	No
Camenzuli et al. (2018)	Coleoptera, Diptera	*Alphitobius diaperinus* and *Hermetia illucens*	*A. diaperinus:* wheat based with apple; *H. illucens:* wheat based and water	Larvae (*A. diapernius*: 2 weeks and *H. illucens*: 1 week)	*A. diapernius*: 14 days; *H. illucens*: 10 days	LC-MS/MS	AFB_1, AFL, AFM_1, AFP_1, AFQ_1; DON, 3-AcDON, 15-AcDON, DON-3G; ZEN, α-ZEN, β-ZEN and OTA	AFB_1: 8, 70 and 390 µg/kg; DON: 3,900, 38,000 and 112,000 µg/kg; ZEN: 280, 2,500 and 13,000 µg/kg; OTA: 170, 1,700 and 1,300 µg/kg. Mixtures: AFB_1: 18, 180 and 430 µg/kg; DON: 4,100, 41,000 and 100,000 µg/kg ; ZEN: 400, 3,800 and 9,400 µg/kg; OTA: 80, 800 and 2,000 µg/kg	No
Cao et al. (2019)	Diptera	*Drosophila simulans*	Grapes	Eggs-adult	Until adult	~~RT-PCR/HRM~~Not mentioned	OTA	OTA: 0.05, 0.5 µg/kg	Yes
Chinnici et al. (1979)	Diptera	*Drosophilia melanogaster* (strains A-9 and A-11)	Control media and media containing AFB1 (prepared according to Chinnici et al. 1976)	Larvae (1-5 days)	Until adult	Thin layer chromatography	AFB_1	AFB_1: 440 and 880 µg/kg	No

Cito et al. (2016)	Coleoptera	*Tenebrio molitor*	Bread and bran	Larvae (mature)	20 days	LC-UV-MS	Beauvericin A, bassianolide and beauverolides	Not specified	*Beauveria bassiana* (strains: B 13/I03, B 13/I11, B 13/I49, B 13/I57, B 13/I63, and B 13/I64)
Davis & Schiefer (1982)	Coleoptera	*Tenebrio molitor*	Whole wheat supplemented with brewer's yeast (9:1)	Larvae (8.5-11.5 mg)	4 weeks	Micro-Kjeldahl technique and Technicon AutoanalyzerCalculations from known concentration	T-2	T-2: 2,000, 4,000, 8,000, 16,000, 32,000, 64,000 and 128,000 µg/kg	No
De Zutter et al. (2016)	Hemiptera, Homoptera	*Sitobion avenae, Acyrthosiphon pisum*	Wheat seedlings and young broad bean plants	Nymphs	3 days	LC-MS/MS	DON, DON-3G, 3- and 15-AcDON	DON: 500, 1,000, 3,000 µg/kg; DON-3G: 500, 1,000, 3,000 µg/kg	No
Dowd (1989)	Lepidoptera	*Heliothis zea*	Pinto bean-based diet + three allelochemicals (gossypol, a saponin, and 6-methoxy2-benzoxazolinone)	Larvae (neonate)	7 days	Observations (mortality, weight) Calculations from known concentration	Fusaric acid	Fusaric acid: 25,000 and 250,000 µg/kg	No

Dowd (1990)	Lepidoptera	*Heliothis zea, Spodoptera frugiperda*	Pinto bean	Larvae (last instar)	48 hours	Enzyme assays (midgut)Thin layer chromatography	DON, T-2, diacetoxyscirpenol	DON: 2,500, 25,000, 250,000 µg/kg; T-2: 2,500, 25,000 µg/kg; diacetoxyscirpenol: 2,500, 25,000 µg/kg	No
Dowd (1993)	Lepidoptera	*Helicoverpa zea, Spodoptera frugiperda*	Pinto bean	Larvae (1st and 3rd instar)	7 days	HPLC	Griseofulvin	Griseofulvin: 25,000 µg/kg	No
Dowd et al. (1988)	Lepidoptera	*Heliothis zea, Spodoptera frugiperda*	Pinto bean	Larvae (neonate)	7 days	Observations (mortality, weight)Not mentioned	Dihydroxyaflavinine, roseotoxin B, penitrem A, verruculogen, cytochalasin H, paspaline, paxilline	Up to 25,000 µg/kg ww (100,000 µg/kg dw) of dihydroxyaflavinine, roseotoxin B, penitrem A, verruculogen, cytochalasin H, paspaline or paxilline	No
Elzaki et al. (2019)	Lepidoptera	*Helicoverpa armigera*	Artificial diet + and - MeJA (prepared according to Qi et al. 2000)	Larvae (2nd instar)	Until pupation	qRT-PCRNot mentioned	AFB_1	AFB_1: 1,000 µg/kg	No
Gulsunoglu et al. (2019)	Diptera	*Hermetia illucens*	SSF kernels	Larvae (2nd instar)	12 days	LC–MS/MS,Observations (mortality, weight), Gravimetric method, Micro-Kjeldahl, Goldfish method	DON	DON: 630-3,580 µg/kg	Fusarium spp.

Gunst et al. (1982)	Diptera	*Drosophilia melanogaster* (strains Florida-9 and Lausanne-S)	Five types: control, media + AFB1, media + AFB1, media + AFG1 and media + ST (prepared according to Chinnici et al., 1976)	Egg-adult	Whole life-cycle	Thin layer chromatography	AFB$_1$, AFB$_2$, AFG$_1$; sterigmatocystin	AFB$_1$, AFB$_2$, AFG$_1$; sterigmatocystin: 200, 600, 2,000 and 4,000 µg/kg	No
Hegde et al. (1967)	Lepidoptera	*Corcyra cephalonica*	Wheat bran, groundnut meal	Larvae (10 days)	20 days	~~Observations (mortality, weight, growth),~~ Paper chromatography	AFB$_1$, AFG$_1$	AFB$_1$: 1,000,000 µg/kg	*Aspergillus flavus*, *Aspergillus oryzae*, *Penicillium purpurogenus* and *Penicillium rubrum*
Jankovic-Tomanic et al. (2019)	Coleoptera	*Tenebrio molitor*	Wheat bran artificially contaminated	Larvae (2 months)	2 weeks	~~Spectrophotometry~~Calculations from known concentration	DON	DON: 4,900, 8,000, 16,000 and 25,000 µg/kg	No
Johnson et al. (2012)	Hymenoptera	*Apis mellifera*	Bee candy (2 sucrose:1 water)	Newly emerged bees	3 days	~~Longevity assay, RNA extraction, Blot analysis, Gut dissection~~Calculations from known concentration	AFB$_1$	AFB$_1$: 20,000 µg/kg	No
Kanaoka et al. (1978)	Lepidoptera	*Bombyx mori*	Artificial diet	Larvae (3rd instar)	8 days	~~PMR spectra, Observations~~	Beauvericin, bassianolide	Beauvericin: 4,000, 8,000, 1,000,000 µg/kg;	*Beauveria bassiana*,

						~~(mortality, weight)~~Silica gel column chromatography		bassianolide: 1,000, 2,000, 4,000, 8,000, 12,000 or 16,000 µg/kg	*Verticillium lecanii*
Kirk et al. (1971)	Diptera	*Drosophilia melanogaster* (strain Oregon R)	Yeast-corn meal molasses growth medium	Larvae (1st instar)	24 days	~~Observations (development)~~] Not mentioned	AFB_1	AFB_1: 10,000 µg/kg	No
Lee & Campbell (2000)	Lepidoptera	*Amyelois transitella*, *Cydia pomonella*	*Am. transitella*: walnuts; *C. pomonella*: apples	Larvae (1st instar)	Not applicable	HPLC w Fluorescence Detector	AFB_1, AFB_2, AFB_{2a}, AFL, AFM_1, AFM_2, AFG_1, AFG_2, AFB_1-8,9-epoxide and AFB_1-8,9-epoxide-GSH conjugate	AFB_1: 3,123,000 µg/kg	No
Leni et al. (2019)	Coleoptera, Diptera	*Alphitobius diaperinus*, *Hermetia illucens*	Naturally contaminated by-products of corn, wheat, rice, rapeseed, apple, olive and carrots	Larvae (2 days)	*A. diaperinus*: 28 days; *H. illucens*: 15 days	HPLC w Fluorescence Detector, UHPLC-MS/MS	DON, 3-AcDON, fusarenone X, diacetoxyscirpenol, T-2, HT-2, FB_1, FB_2, ZEN, AFB_1, patulin, OTA, nivalenol	DON: 416, 468, 557, 608, 726, 755 µg/kg; FB_1: 127 µg/kg; FB_2: <LOD; ZEN: <LOD	No

Llewellyn et al. (1988)	Hemiptera	*Oncopeltus fasciatus*	Water, unsalted raw sunflower seeds	Larvae (5th instar)	21 days	~~Observations (mortality, weight)~~Calculations from known concentration	AFB_1	AFB_1: 5,000 µg/kg	No
Llewellyn et al. (1976)	Blattodea	*Paradelphomyia americana*	Sucrose water	Adult (males)	56 days	~~Observations (weight)~~Not mentioned	AFB_1	AFB_1: 12,000 µg/kg	No
Matsumura & Knight (1967)	Diptera	*Aedes aegypti*, *Drosophila melanogaster* and *Musca domestica*	*Ae. aegypti*: distilled water; *D. melanogaster*: standard food medium; *M. domestica*: sucrose solution and milk (1:1)	*Ae. aegypti*: larvae (4th instar) and adults; *D. melanogaster*: adults; *M. domestica*: adults	5 days	~~Observations (mortality)~~Not mentioned	AFB_1	AFB_1: *Ae. aegypti* larvae 3,000 µg/kg (unclear for *Ae. aegypti* adults, *D. melanogaster* and *M. domestica*)	No
Meijer et al. (2019)	Diptera	*Hermetia illucens*	Wheat based mashed feed	Larvae (1st instar)	9 days	LC-MS~~, S9 Fractions, Observations (mortality, weight)~~	AFB_1	AFB_1: 500 µg/kg	No
Melone & Chinnici (1986)	Diptera	*Drosophilia melanogaster* (strains Oregon-R and Lausanne-S)	Fly culture medium (dextrose, yeast, agar, inorganic salts, and methyl p-hydroxybenzoate)	Egg-adult	Whole life-cycle, multiple generations	~~Observations (mortality)~~Calculations from known concentration	AFB_1	AFB_1: 500, 1,000, 1,300, 1,600, 1,900, 2,200 and 2,500 µg/kg	No

Mencarelli et al. (2013)	Lepidoptera	*Ostrinia nubilalis*	Corn	Larvae (4th instar)	14 days	~~Observations (mortality)~~Not mentioned	AFB_1	AFB_1: 125-30,000 µg/kg	*Aspergillus flavus*
Miller et al. (2008)	Lepidoptera	*Choristoneura fumiferana*	Spruce Trees	Larvae (2nd instar)	3 months (until 6th instar)	~~Observations (weight)~~Not mentioned	Rugulosin	Rugulosin: 850 µg/kg (geometric mean)	No
Nevins & Grant (1971)	Diptera	*Musca domestica*	Moistened dog food pellets	Larvae (2nd instar)	7 days	Thin layer chromatography	AFB_1	AFB_1: 20 µg/kg	*Aspergillus flavusoryzae*
Niermans et al. (2019)	Coleoptera	*Tenebrio molitor*	Wheat flour	Larvae (42 days)	4 or 8 weeks	HPLC-MS/MS, ~~Observations (mortality, weight)~~	DON, ZEN, β-ZEL	DON: spiked with 568 and 576 µg/kg, artificially contaminated with 939 and 2,101 µg/kg and naturally contaminated with 2,854 and 4,588 µg/kg. ZEN: spiked with 589 and 2,254 µg/kg, artificially contaminated with 427 and 2,283 µg/kg and naturally contaminated with 602 and 919 µg/kg	No
Niu et al. (2011)	Hymenoptera	*Apis mellifera*	Bee candy (2 sucrose:1 water)	Workers	3 days	~~Bioassays (mortality)~~Calculations from known concentration	AFB_1, OTA	AFB_1: 500, 1,000, 2,000, 5,000, 7,000, 10,000, 15,000, 20,000 µg/kg.	No

								OTA: 1,000, 5,000, 10,000, 20,000, 40,000, 60,000 µg/kg	
Niu et al. (2009)	Lepidoptera	*Amyelois transitella* and *Helicoverpa zea*	Not specified	Larvae (1st and 5th instar)	Until pupation	HPLC w photodiode array UV detector	AFB_1, OTA	AFB_1: 1,000, 5,000, 10,000, 20,000, 50,000 and 100,000 µg/kg. OTA: 1,000, 5,000, 10,000, 20,000 and 50,000 µg/kg	No
Niu et al. (2008)	Lepidoptera	*Helicoverpa zea*	Artificial diet	Larvae (5th instar)	48 hours	HPLC, w UV detector, LC-MS, RT-PCR	AFB_1	AFB_1: 1,000 µg/kg	No
Ochoa Sanabria et al. (2019)	Coleoptera	*Tenebrio molitor*	Wheat (naturally contaminated)	Larvae (7th-9th instar)	Until pupation	HPLC-MS	DON, 3-AcDON	DON: 2,000, 10,000, 12,000 µg/kg. 3-AcDON 52, 63, 205 µg/kg	No
Ohtomo et al. (1975)	Lepidoptera	*Bombyx mori*	Not specified	Larvae (5th instar)	5 days	Observations (mortality), Thin layer chromatographyLC, ultra-violet and infrared analysis	AFB_1	AFB_1: 15,614 µg/kg	*Aspergillus flavus* (K-199)
Patterson et al. (1987)	Diptera, Lepidoptera	*Drosophila melanogaster, Spodoptera littoralis*	*D. melanogaster*: maize; *S.littoralis:* beans	Larvae *(D. melanogaster:* 2nd instar and *S. littoralis:* 4th instar)	3 days	Observations (mortality)Thin layer chromatography	OTA, brevianamide A, citrinin, penicillic acid, viomellein,	10,000 µg/kg for all	*Penicillium*

							patulin, cyclopenol		
Patterson et al. (1990)	Lepidoptera	*Spodoptera frugiperda* and *Heliothis virescens*	Bean based diet	Larvae (3rd-6th instar)	3 days	~~Observations (mortality, weight)~~Preparative layer chromatography	Brevianamide A and D, OTA	Brenianamide A and B, OTA: 1,000 and 10,000 µg/kg	*Penicillium vinidicatum*
Piacenza et al. (2020)	Coleoptera	*Tenebrio molitor*	Oat flakes	larvae (42 days)	4 weeks	HPLC-MS/MS~~, Observations (mortality, weight)~~	T-2 and HT-2	Sum of T-2 and HT-2 (approx. 100 and 250 µg/kg)	No
Purschke et al. (2017)	Diptera	*Hermetia illucens*	Substrates based on corn semolina + contaminated corn grains (DON and ZEN)	Larvae (7 days)	13 days	HPLC-MS/MS QTRAP	AFB_1, AFB_2, AFG_2, OTA, DON, ZEN	DON: naturally contaminated with 4600 µg/kg, AFB_1: 88 µg/kg, AFB_2: 17 µg/kg, AFG_2: 46 µg/kg, OTA: 260 µg/kg ZEN: naturally contaminated with 860 µg/kg	No
Rizwan-UL-Haq et al. (2009)	Lepidoptera	*Spodoptera exigua*	Semi-synthetic diet	Larvae (neonate)	8 days	~~Observations (mortality, weight)~~HPLC	Destruxin B	Destruxin B: 15,000, 30,000, 45,000, 60,000, 75,000, 90,000 µg/kg	*Metarhizium anisoplae*
Sadek (1996)	Lepidoptera	*Spodoptera littoralis*	Artificial diet	Larvae (2nd instar)	Whole life-cycle	~~Observations (mortality, development)~~Not mentioned	AFB_1, AFB_2, AFG_1	AFB_1: 500, 1,000, 2,000, 2,500, 3,000 and 3,500 µg/kg, AFB_2: 2,000, 3,000 and 4,000 µg/kg,	No

								AFG_1: 1,000, 2,000, 3,000 and 4,000 µg/kg	
Saner et al. (1996)	Diptera	*Drosophila melanogaster* (strain Oregon R(R))	Not specified	-	Not applicable	~~Northern blot analysis~~Not mentioned	AFB_1	Not specified	No
Şişman et al. (2006)	Diptera	*Drosophila melanogaster* (strain Oregon-R)	Yeast-agar-sugar medium	Eggs, Larvae, Pupae	37-58 days	~~Observations (development)~~Not mentioned	AFB_1	AFB_1: 200, 500, 800 µg/kg	No
Sree & Padmaja (2008)	Lepidoptera	*Spodoptera litura*	Artificial diet	Larvae (9 days)	1, 24, 48 hours	~~Observations (mortality, weight)~~HPLC	Destruxin	Destruxin: 88-693 µg/kg	*Metarhizium anisopliae* (M-10) & (M-19)
Sumarah et al. (2008)	Lepidoptera	*Choristoneura fumiferana*, *Lambdina fiscellaria*, and *Zeiraphera canadensis*	*Picea glauca* seedlings	Larvae (2nd and 3rd instar)	7 days	~~Observations (weight)~~LC-MS	Rugulosin	Rugulosin: 2,713-81,375 µg/kg	*Aspergillus fumigatus*
Van Broekhoven et al. (2014)	Coleoptera	*Tenebrio molitor*, *Zophobas atratus*, and *Alphitobius diaperinus*	Maize, beer yeast, bread remains, pent grains, potato steam peelings, cookie remains	Larvae (neonate)	*T. molitor:* 26 days; *Z. atratus*: 33 days; *A. diaperinus*: 21 days	LC-MS	T-2, ZEN, OTA	T-2, ZEN, OTA: 500 µg/kg	No

Van Broekhoven et al. (2017)	Coleoptera	*Tenebrio molitor*	Wheat flour (spiked and naturally contaminated)	Larvae (5 weeks)	14 days	LC-MS	DON	DON: naturally contaminated with 4,900 µg/kg, spiked with: 8,000 µg/kg	No
Wright et al. (1976)	Coleoptera	*Tribolium confusum*	Whole wheat flour plus 5% brewer's yeast	Larvae	60 days	Observations (mortality, weight, fecundity)Not mentioned	T-2	T-2: 10,000, 100,000 µg/kg	No
Zeng et al. (2006)	Lepidoptera	*Helicoverpa zea*	Semi-synthetic diet containing wheat germ	Larvae (1st, 3rd and 5th instars)	13 days	Bioassays (mortality, growth)Calculations from known concentration	AFB_1	AFB_1: 1, 20, 200, 1,000, 20,000 µg/kg	No
Zeng et al. (2013)	Lepidoptera	*Trichoplusia ni*	Semi-synthetic diet containing wheat germ	Larvae (1, 5, 7, 10 days)	6-16 days	Bioassays (mortality, growth)Calculations from known concentration	AFB_1	AFB_1: 1, 20, 200, 1,000, 3,000, 5,000 µg/kg	No
Zeng et al. (2009)	Lepidoptera	*Helicoverpa zea*	Semi-synthetic diet containing wheat germ	Larvae (4th and 5th instar)	12 days	Bioassays, RT-PCR, Transcript analysisCalculations from known concentration	AFB_1	AFB_1: 1,000 µg/kg	No
Zhao et al. (2018)	Coleoptera	*Ahasverus advena*	Wheat flour, rolled oats and yeast (5:5:1 by weight)	Larvae (1, 5 ,10 days old)	4-14 days	Observations (mortality, growth)Calculations	AFB_1	AFB_1: 0, 500,000, 1,000,000, 2,000,000, 4,000,000, 8,000,000, and 16,000,000 µg/kg	No

						from known<br><br>concentration			
