# Supplementary material for: A systematic literature review on the effects of mycotoxin exposure on insects and on mycotoxin accumulation and biotransformation
Source: Mycotoxin Res. 2021 Oct 7;37(4):279–95. doi: 10.1007/s12550-021-00441-z (PMC8571154; doi:10.1007/s12550-021-00441-z)
Supplement: Supplementary file 4 — Supplementary file4 Table S2: Overview of data on mortality after AFB1 exposure for insects in the orders Diptera, Coleoptera, Lepidoptera and species Apis mellifera. (PDF 184 KB) [file 12550_2021_441_MOESM4_ESM.pdf]

Mycotoxin Research

A Systematic Literature Review on the Effects of Mycotoxin Exposure on Insects, and on Mycotoxin Accumulation and Biotransformation

K. Niermans[1,2], A.M. Meyer[2], E.F. Hoek- van den Hil[2], J.J.A. van Loon[1], H.J. van der Fels-Klerx[2]

[1] Wageningen University, Department of Plant Sciences, Laboratory of Entomology, Wageningen, The Netherlands

[2] Wageningen Food Safety Research, Akkermaalsbos 2, 6708 WB, Wageningen, The Netherlands

Corresponding Author: ine.vanderfels@wur.nl

Supplementary Table S2: Overview of data on mortality after AFB_1 exposure for insects in the orders Diptera, Coleoptera, Lepidoptera and specie *Apis mellifera*

Species	Conc. of AFB1	Unit	Conc. shown in graph (µg/kg)	Start amount of larvae	Mortality/Survival mentioned in study (%)	Mortality in graph (%)	Age	Exposure time	Reference
Diptera									

Drosophila melanogaster (strain A-9)	0	ppm	0	25	23.67 Adults	5	Eggs	Until adult	Chinnici et al. (1979)
Drosophila melanogaster (strain A-9)	0.44	ppm	440	25	0 Adults	100	Eggs	Until adult	Chinnici et al. (1979)
Drosophila melanogaster (strain A-9)	0.88	ppm	880	25	0 Adults	100	Eggs	Until adult	Chinnici et al. (1979)
Drosophila melanogaster (strain A-11)	0	ppm	0	25	22.67 Adults	9	Eggs	Until adult	Chinnici et al. (1979)
Drosophila melanogaster (strain A-11)	0.44	ppm	440	25	22.83 Adults	9	Eggs	Until adult	Chinnici et al. (1979)
Drosophila melanogaster (strain A-11)	0.88	ppm	880	25	19.67 Adults	21	Eggs	Until adult	Chinnici et al. (1979)
Drosophila melanogaster (strain Florida 9)	0	ppm	0	25	15.67 Adults	37	Eggs	Until adult	Gunst et al. (1982)
Drosophila melanogaster (strain Florida 9)	0.2	ppm	200	25	8.5 Adults	66	Eggs	Until adult	Gunst et al. (1982)
Drosophila melanogaster (strain Florida 9)	0.6	ppm	600	25	0.17 Adults	99	Eggs	Until adult	Gunst et al. (1982)

Drosophila melanogaster (strain Florida 9)	2	ppm	2,000	25	0 Adults	100	Eggs	Until adult	Gunst et al. (1982)
Drosophila melanogaster (strain Florida 9)	4	ppm	4,000	25	0 Adults	100	Eggs	Until adult	Gunst et al. (1982)
Drosophila melanogaster (strain Laussane-S)	0	ppm	0	25	17.83 Adults	29	Eggs	Until adult	Gunst et al. (1982)
Drosophila melanogaster (strain Laussane-S)	0.2	ppm	200	25	22.67 Adults	9	Eggs	Until adult	Gunst et al. (1982)
Drosophila melanogaster (strain Laussane-S)	0.6	ppm	600	25	16 Adults	36	Eggs	Until adult	Gunst et al. (1982)
Drosophila melanogaster (strain Laussane-S)	2	ppm	2,000	25	0.33 Adults	99	Eggs	Until adult	Gunst et al. (1982)
Drosophila melanogaster (strain Laussane-S)	4	ppm	4,000	25	0 Adults	100	Eggs	Until adult	Gunst et al. (1982)
Drosophila melanogaster (strain Oregon-R)	10	ppm	10,000	x	100 Adults	100	Eggs	8 days	Kirk et al. (1971)

Drosophila melanogaster (intercrossed strain Oregon-R & Laussane-S)	0	ppm	0	25	21	21	Eggs	Until adult	Melone & Chinnici (1986)
Drosophila melanogaster (intercrossed strain Oregon-R & Laussane-S)	0.5	ppm	500	25	16	16	Eggs	Until adult	Melone & Chinnici (1986)
Drosophila melanogaster (intercrossed strain Oregon-R & Laussane-S)	1	ppm	1,000	25	46, 54	50	Eggs	Until adult	Melone & Chinnici (1986)
Drosophila melanogaster (intercrossed strain Oregon-R & Laussane-S)	1.3	ppm	1,300	25	82, 64, 90	79	Eggs	Until adult	Melone & Chinnici (1986)
Drosophila melanogaster (intercrossed strain Oregon-R & Laussane-S)	1.6	ppm	1,600	25	99, 100, 95, 88	96	Eggs	Until adult	Melone & Chinnici (1986)
Drosophila melanogaster (intercrossed strain Oregon-R & Laussane-S)	1.9	ppm	1,900	25	98, 98, 100	99	Eggs	Until adult	Melone & Chinnici (1986)

Drosophila melanogaster (intercrossed strain Oregon-R & Laussane-S)	2.2	ppm	2,200	25	98, 97, 97, 99	98	Eggs	Until adult	Melone & Chinnici (1986)
Drosophila melanogaster (intercrossed strain Oregon-R & Laussane-S)	2.5	ppm	2,500	25	100, 100, 100	100	Eggs	Until adult	Melone & Chinnici (1986)
Hermetia illucens	0	mg/kg ww	0	100	2	2	One-week old larvae	10 days	Camenzuli et al. (2018)
Hermetia illucens	0.008	mg/kg ww	8	100	0	0	One-week old larvae	10 days	Camenzuli et al. (2018)
Hermetia illucens	0.07	mg/kg ww	70	100	2	3	One-week old larvae	10 days	Camenzuli et al. (2018)
Hermetia illucens	0.39	mg/kg ww	390	100	2	2	One-week old larvae	10 days	Camenzuli et al. (2018)
Hermetia illucens	0	mg/kg	0	100	1	1	24 h	9 days	Meijer et al. (2019)
Hermetia illucens	0.5	mg/kg	500	100	3	3	24 h	9 days	Meijer et al. (2019)
Hermetia illucens	0	mg/kg	0	100	8	8	24 h	10 days	Bosch et al. (2017)
Hermetia illucens	0.1	mg/kg	100	100	9	9	24 h	10 days	Bosch et al. (2017)
Hermetia illucens	0.2	mg/kg	200	100	6	6	24 h	10 days	Bosch et al. (2017)
Hermetia illucens	0.4	mg/kg	400	100	9	9	24 h	10 days	Bosch et al. (2017)

Aedes aegypti	0	ppm	0	100	2	2	Freshly emerged	5 days	Matsumara & Knight (1967)
Aedes aegypti	3	ppm	3,000	100	2	2	Freshly emerged	5 days	Matsumara & Knight (1967)
Coleoptera									
Tenebrio Molitor	0	mg/kg	0	100	8	8	First instar	40 days	Bosch et al. (2017)
Tenebrio Molitor	0.1	mg/kg	100	100	3	3	First instar	40 days	Bosch et al. (2017)
Tenebrio Molitor	0.2	mg/kg	200	100	2	2	First instar	40 days	Bosch et al. (2017)
Tenebrio Molitor	0.4	mg/kg	400	100	3	3	First instar	40 days	Bosch et al. (2017)
Alphitobius diaperinus	0	mg/kg ww	0	200	26	26	Two-week old larvae	14 days	Camenzuli et al. (2018)
Alphitobius diaperinus	0.008	mg/kg ww	8	200	19	19	Two-week old larvae	14 days	Camenzuli et al. (2018)
Alphitobius diaperinus	0.07	mg/kg ww	70	200	17	17	Two-week old larvae	14 days	Camenzuli et al. (2018)
Alphitobius diaperinus	0.39	mg/kg ww	390	200	21	21	Two-week old larvae	14 days	Camenzuli et al. (2018)
Ahasverus advena	0	ppm	0	25	16	16	One-day old	4 days	Zhao et al. (2018)
Ahasverus advena	500	ppm	500,000	25	29	29	One-day old	4 days	Zhao et al. (2018)
Ahasverus advena	1,000	ppm	1,000,000	25	44	44	One-day old	4 days	Zhao et al. (2018)
Ahasverus advena	2,000	ppm	2,000,000	25	52	52	One-day old	4 days	Zhao et al. (2018)

Ahasverus advena	4,000	ppm	4,000,000	25	84	84	One-day old	4 days	Zhao et al. (2018)
Ahasverus advena	8,000	ppm	8,000,000	25	99	99	One-day old	4 days	Zhao et al. (2018)
Ahasverus advena	0	ppm	0	25	19	19	Five-days-old	9 days	Zhao et al. (2018)
Ahasverus advena	500	ppm	500,000	25	20	20	Five-days-old	9 days	Zhao et al. (2018)
Ahasverus advena	1,000	ppm	1,000,000	25	39	39	Five-days-old	9 days	Zhao et al. (2018)
Ahasverus advena	2,000	ppm	2,000,000	25	49	49	Five-days-old	9 days	Zhao et al. (2018)
Ahasverus advena	4,000	ppm	4,000,000	25	75	75	Five-days-old	9 days	Zhao et al. (2018)
Ahasverus advena	8,000	ppm	8,000,000	25	87	87	Five-days-old	9 days	Zhao et al. (2018)
Ahasverus advena	16,000	ppm	16,000,000	25	93	93	Five-days-old	9 days	Zhao et al. (2018)
Ahasverus advena	0	ppm	0	25	17	17	Ten-days-old	14 days	Zhao et al. (2018)
Ahasverus advena	500	ppm	500,000	25	19	19	Ten-days-old	14 days	Zhao et al. (2018)
Ahasverus advena	1,000	ppm	1,000,000	25	31	31	Ten-days-old	14 days	Zhao et al. (2018)
Ahasverus advena	2,000	ppm	2,000,000	25	33	33	Ten-days-old	14 days	Zhao et al. (2018)
Ahasverus advena	4,000	ppm	4,000,000	25	41	41	Ten-days-old	14 days	Zhao et al. (2018)
Ahasverus advena	8,000	ppm	8,000,000	25	47	47	Ten-days-old	14 days	Zhao et al. (2018)
Ahasverus advena	160,000	ppm	160,000,000	25	57	57	Ten-days-old	14 days	Zhao et al. (2018)
Lepidoptera									

Ostrinia nubilalis	2.08	log ng/g	120	15	20	20	Fourth instar	14 days	Mencarelli et al. (2013)
Ostrinia nubilalis	3.02	log ng/g	1,047	15	40	40	Fourth instar	14 days	Mencarelli et al. (2013)
Ostrinia nubilalis	4	log ng/g	10,000	15	65	65	Fourth instar	14 days	Mencarelli et al. (2013)
Ostrinia nubilalis	4.7	log ng/g	50,119	15	70	70	Fourth instar	14 days	Mencarelli et al. (2013)
Trichoplusia ni	1	µg/g	1,000	20	90	90	First instar	10 days	Zeng et al. (2013)
Trichoplusia ni	1	µg/g	1,000	20	100	100	First instar	16 days	Zeng et al. (2013)
Spodoptera littoralis	0.00	ppm	0	60	7	7	Second instar	Until pupation	Sadek (1996)
Spodoptera littoralis	0.50	ppm	500	60	13	13	Second instar	Until pupation	Sadek (1996)
Spodoptera littoralis	1.00	ppm	1,000	60	6	6	Second instar	Until pupation	Sadek (1996)
Spodoptera littoralis	2.00	ppm	2,000	60	19	19	Second instar	Until pupation	Sadek (1996)
Spodoptera littoralis	2.50	ppm	2,500	60	27	27	Second instar	Until pupation	Sadek (1996)

Spodoptera littoralis	3.00	ppm	3,000	60	33	33	Second instar	Until pupation	Sadek (1996)
Amyelois transitella	0.00	µg/g	0	20	10	10	First instar	48 h	Niu et al. (2009)
Amyelois transitella	1.00	µg/g	1,000	20	10	10	First instar	48 h	Niu et al. (2009)
Amyelois transitella	5.00	µg/g	5,000	20	15	15	First instar	48 h	Niu et al. (2009)
Amyelois transitella	10.00	µg/g	10,000	20	20	20	First instar	48 h	Niu et al. (2009)
Helicoverpa Zea	0.00	ng/g	0	20	2	2	First instar	48 h	Niu et al. (2009)
Helicoverpa Zea	10.00	ng/g	10	20	5	5	First instar	48 h	Niu et al. (2009)
Helicoverpa Zea	20.00	ng/g	20	20	3	3	First instar	48 h	Niu et al. (2009)
Helicoverpa Zea	40.00	ng/g	40	20	15	15	First instar	48 h	Niu et al. (2009)
Helicoverpa Zea	60.00	ng/g	60	20	50	50	First instar	48 h	Niu et al. (2009)
Helicoverpa Zea	80.00	ng/g	80	20	75	75	First instar	48 h	Niu et al. (2009)
Helicoverpa Zea	100.00	ng/g	100	20	85	85	First instar	48 h	Niu et al. (2009)
Helicoverpa Zea	120.00	ng/g	120	20	85	85	First instar	48 h	Niu et al. (2009)
Helicoverpa Zea	140.00	ng/g	140	20	90	90	First instar	48 h	Niu et al. (2009)
Helicoverpa Zea	0.00	ng/g	0	20	20	20	First instar	15 days	Zeng et al. (2006)
Helicoverpa Zea	1.00	ng/g	1	20	37	37	First instar	15 days	Zeng et al. (2006)

Helicoverpa Zea	20.00	ng/g	20	20	45	45	First instar	15 days	Zeng et al. (2006)
Helicoverpa Zea	200.00	ng/g	200	20	100	100	First instar	15 days	Zeng et al. (2006)
Helicoverpa Zea	1.00	ug/g	1,000	20	100	100	First instar	15 days	Zeng et al. (2006)
Helicoverpa Zea	0.00	ng/g	0	20	0	0	Third instar	21 days	Zeng et al. (2006)
Helicoverpa Zea	20.00	ng/g	20	20	0	0	Third instar	21 days	Zeng et al. (2006)
Helicoverpa Zea	200.00	ng/g	200	20	27	27	Third instar	21 days	Zeng et al. (2006)
Helicoverpa Zea	1.00	µg/g	1,000	20	100	100	Third instar	21 days	Zeng et al. (2006)
Helicoverpa Zea	20.00	µg/g	20,000	20	100	100	Third instar	21 days	Zeng et al. (2006)
Helicoverpa Zea	0.00	ng/g	0	20	0	0	Fifth instar	10 days	Zeng et al. (2006)
Helicoverpa Zea	20.00	ng/g	20	20	0	0	Fifth instar	10 days	Zeng et al. (2006)
Helicoverpa Zea	200.00	ng/g	200	20	0	0	Fifth instar	10 days	Zeng et al. (2006)
Helicoverpa Zea	1.00	µg/g	1,000	20	10	10	Fifth instar	10 days	Zeng et al. (2006)
Helicoverpa Zea	20.00	µg/g	20,000	20	100	100	Fifth instar	10 days	Zeng et al. (2006)
Helicoverpa armigera	1.00	µg/g	1,000	20	40	40	Fourth instar	6 days	Elzaki et al. (2019)
Helicoverpa armigera	1.00	µg/g	1,000	20	55	55	Fourth instar	9 days	Elzaki et al. (2019)
Other									
Apis mellifera	0.00	µg/g	0	30	5	5	Newly enclosed adults	72 h	Niu et al. (2011)

Apis mellifera	0.50	µg/g	500	30	5	5	Newly enclosed adults	72 h	Niu et al. (2011)
Apis mellifera	1.00	µg/g	1,000	30	30	30	Newly enclosed adults	72 h	Niu et al. (2011)
Apis mellifera	2.50	µg/g	2,500	30	20	20	Newly enclosed adults	72 h	Niu et al. (2011)
Apis mellifera	5.00	µg/g	5,000	30	35	35	Newly enclosed adults	72 h	Niu et al. (2011)
Apis mellifera	7.00	µg/g	7,000	30	50	50	Newly enclosed adults	72 h	Niu et al. (2011)
Apis mellifera	10.00	µg/g	10,000	30	90	90	Newly enclosed adults	72 h	Niu et al. (2011)
Apis mellifera	15.00	µg/g	15,000	30	100	100	Newly enclosed adults	72 h	Niu et al. (2011)
Apis mellifera	20.00	µg/g	20,000	30	100	100	Newly enclosed adults	72 h	Niu et al. (2011)
