# Supplementary material for: A systematic literature review on the effects of mycotoxin exposure on insects and on mycotoxin accumulation and biotransformation
Source: Mycotoxin Res. 2021 Oct 7;37(4):279–95. doi: 10.1007/s12550-021-00441-z (PMC8571154; doi:10.1007/s12550-021-00441-z)
Supplement: Supplementary file 5 — Supplementary file5 Table S3: Concentrations of parent compounds and metabolites in initial substrate, larvae, and residual material with mass balance provided of Hermetia illucens, Tenebrio molitor, and Alphitobius diaperinus. (PDF 341 KB) [file 12550_2021_441_MOESM5_ESM.pdf]

Mycotoxin Research

A Systematic Literature Review on the Effects of Mycotoxin Exposure on Insects, and on Mycotoxin Accumulation and Biotransformation

K. Niermans[1,2], A.M. Meyer[2], E.F. Hoek- van den Hil[2], J.J.A. van Loon[1], H.J. van der Fels-Klerx[2]

[1] Wageningen University, Department of Plant Sciences, Laboratory of Entomology, Wageningen, The Netherlands

[2] Wageningen Food Safety Research, Akkermaalsbos 2, 6708 WB, Wageningen, The Netherlands

Corresponding Author: ine.vanderfels@wur.nl

Supplementary Table S3: Concentrations of parent compounds and metabolites in initial substrate, larvae, and residual material with mass balance provided of *Hermetia illucens*, *Tenebrio molitor*, and *Alphitobius diaperinus*

Hermetia illucens											
Parent compound	Type of contamination	Initial Dose (µg/kg)	Initial Conc. in Feed (µg/kg)	Conc. in Larvae (µg/kg)	Conc. in Residue(s) (µg/kg)	Metabolite (s)	Initial Conc. in Feed (µg/kg)	Conc. in Larvae (µg/kg)	Conc. in Residue (µg/kg)	Total Calculated Mass Balance %	Reference

AFB_1	S	13	0.231	<LOD	0.010	AFM_1		<LOD		4 [1]	Bosch et al. (2017)
	S	23	0.419	<LOD	0.032	AFM_1		<LOD		8 [1]	
	S	49	0.875	<LOD	0.047	AFM_1		<LOD		5 [1]	
	S	84	1.5	<LOD	0.110	AFM_1		<LOD		7 [1]	
	S	204	3.7	<LOD	0.275	AFM_1		<LOD		7 [1]	
	S	415	7.5	<LOD	1.3	AFM_1		<LOD		17 [1]	
AFB_1	S	13	13.3	<LOD	10.9	AFB_2	2.6	<LOD	<LOQ	48 [2]	Purschke et al. (2017)
						AFG_2	7	<LOD	<LOQ		
DON	S	698	697.7	<LOD	1,135.7					163 [2]	
OTA	S	39	39.4	<LOD	<LOQ					0 [2]	
ZEN	S	130	130.7	<LOD	103.7					79 [2]	
AFB_1	S	20 (ww)	8 (ww)	<LOQ	5.5 + <LOQ(dw)	AFM_1		<LOQ	<LOQ	11 [1]	Camenzuli et al. (2018)
						AFL		<LOQ	<LOQ		
						AFP_1		<LOQ	<LOQ		
						AFQ_1		<LOQ	<LOQ		
	S	200 (ww)	70 (ww)	<LOQ	62 + 1.8 (dw)	AFM_1		<LOQ	<LOQ	18 [1]	
						AFL		<LOQ	<LOQ		
						AFP_1		<LOQ	<LOQ		
						AFQ_1		<LOQ	<LOQ		

	S	500 (ww)	390 (ww)	<LOQ	303.3 + 5.3 (dw)	AFM_1		<LOQ	<LOQ	16 [1]	
						AFL		<LOQ	<LOQ		
						AFP_1		<LOQ	<LOQ		
						AFQ_1		<LOQ	<LOQ		
AFB_1 (Mix)	S	20 (ww)	18 (ww)	<LOQ	13.7 + <LOQ(dw)	AFM_1		<LOQ	<LOQ	16 [1]	
						AFL		<LOQ	<LOQ		
						AFP_1		<LOQ	<LOQ		
						AFQ_1		<LOQ	<LOQ		
	S	200 (ww)	180 (ww)	<LOQ	156.7 + 1.8 (dw)	AFM_1		<LOQ	<LOQ	17 [1]	
						AFL		<LOQ	<LOQ		
						AFP_1		<LOQ	<LOQ		
						AFQ_1		<LOQ	<LOQ		
	S	500 (ww)	430 (ww)	<LOQ	353.3 + 4.6 (dw)	AFM_1		<LOQ	<LOQ	16 [1]	
						AFL		<LOQ	67 + <LOQ (dw)		
						AFP_1		<LOQ	<LOQ		
						AFQ_1		<LOQ	<LOQ		

	S	5,000 (ww)	3,900 (ww)	<LOQ	7,700 + 300 (dw)					39 [1]	
DON	S	50,000 (ww)	38,000 (ww)	129.3 (dw)	86,300 + 2,500 (dw)					45 [1]	
	S	125,000 (ww)	112,000 (ww)	256.7 (dw)	316,700 +5,500 (dw)					55 [1]	
	S	5,000 (ww)	4,100 (ww)	<LOQ	15,700 + 200 (dw)					80 [1]	
DON (Mix)	S	50,000 (ww)	41,000 (ww)	109.5 (dw)	150,000 + 1,800 (dw)					74 [1]	
	S	125,000 (ww)	100,000 (ww)	176.7 (dw)	296,700 + 4,200(dw)					55 [1]	
	S	100 (ww)	170 (ww)	<LOQ	400 + 5.1 (dw)					53 [1]	
OTA	S	1,000 (ww)	1,300 (ww)	2.2 (dw)	3,500 +26.3 (dw)					57 [1]	
	S	2,500 (ww)	1,700 (ww)	2.6 (dw)	5,100 + 45.0 (dw)					56 [1]	

OTA (Mix)	S	100 (ww)	80 (ww)	<LOQ	200 + <LOQ(dw)					41 [1]	
	S	1,000 (ww)	800 (ww)	1.8 (dw)	2,500 + 27.3 (dw)					62 [1]	
	S	2,500 (ww)	2,000 (ww)	3.9 (dw)	5,000 + 69.0 (dw)					46 [1]	
ZEN	S	500 (ww)	280 (ww)	<LOQ	700 + <LOQ (dw)	α -ZEL		<LOQ	640 + <LOQ (dw)	104 [1]	
	S					β-ZEL		<LOQ	180 + <LOQ (dw)		
	S	5,000 (ww)	2,500 (ww)	<LOQ	6,600 +183.3 (dw)	α -ZEL		5 (dw)	7,200 + 140 (dw)	128 [1]	
	S					β-ZEL		<LOQ	2,300 + 42 (dw)		
	S	12,500 (ww)	13,000 (ww)	27.5 (dw)	35,300 + 26.3 (dw)	α -ZEL		25 (dw)	37,300 + 600 (dw)	133 [1]	

	S					β-ZEL		7 (dw)	11,200 + 180 (dw)		
ZEN (Mix)	S	500 (ww)	400 (ww)	<LOQ	990 + <LOQ (dw)	α -ZEL		<LOQ	740 + 22 (dw)	100 [1]	
						β-ZEL		<LOQ	200 + <LOQ (dw)		
	S	5,000 (ww)	3,800 (ww)	<LOQ	10,200 + 78.7 (dw)	α -ZEL		11 (dw)	12,700 + 210 (dw)	143 [1]	
						β-ZEL		<LOQ	3,600 + 71 (dw)		
	S	12,500 (ww)	9,400 (ww)	<LOQ	22,700 + 226.7 (dw)	α -ZEL		29 (dw)	28,300 + 630 (dw)	118 [1]	
						β-ZEL		6.7 (dw)	8,600 + 130 (dw)		

DON	NC, CDR		779	<LOD	1,473	3-AcDON	<LOD	<LOD	<LOD	± 81 [1]	Leni et al. (2019)
FB_1	NC, CDR		573	<LOD	951					± 72 [1]	
FB_2	NC, CDR		441	<LOD	344					± 5 [1]	
ZEN	NC, CDR		<LOD	<LOD	334						
Tenebrio Molitor											
Parent compound	Type of contamination	Initial Dose (µg/kg)	Initial Conc. in Feed (µg/kg)	Conc. in Larvae (µg/kg)	Conc. in Residue (µg/kg)	Metabolite (s)	Initial Conc. in Feed (µg/kg)	Conc. in Larvae (µg/kg)	Conc. in Residue (µg/kg)	Total Calculated Mass Balance %	Reference
AFB_1	S	13	0.115	<LOD	0.005	AFM_1		<LOD		4 [1]	Bosch et al. (2017)
	S	23	0.209	<LOD	0.013	AFM_1		0.0017		6 [1]	
	S	49	0.437	0.001	0.035	AFM_1		<LOD		8 [1]	
	S	84	0.756	0.001	0.083	AFM_1		0.0009		11 [1]	
	S	204	1.8	0.002	0.139	AFM_1		0.0009		8 [1]	

	S	415	3.7	0.002	0.352	AFM_1		0.0011		9 [1]	
DON	NC		200	136	131	3-AcDON	<LOD	66	286	234 [2]	Sanabria et al. (2019)
						15-AcDON	<LOD	<LOD	<LOD		
						Nivalenol [3]	<LOD	<LOD	50		
	NC		2,000	127	324	3-AcDON	63	66	323	31 [2]	
						15-AcDON	<LOD	<LOD	<LOD		
						Nivalenol [3]	<LOD	<LOD	<LOD		
	NC		10,000	122	230	3-AcDON	52	66	326	6 [2]	
						15-AcDON	<LOD	<LOD	<LOD		
						Nivalenol [3]	<LOD	<LOD	51		
	NC		12,000	131	742	3-AcDON	205	65	280	8 [2]	
						15-AcDON	<LOD	<LOD	<LOD		
						Nivalenol [3]	<LOD	<LOD	<LOD		
DON (mix)	NC	2854	99.9	<LOD	51.6					52 [2]	Niermans et al. (2019)
	NC	602.3	21.1	<LOD	4.8	α -ZEL	<LOD	<LOD	1.8	48 [2]	

ZEN (mix)						β-ZEL	0.2	<LOD	3.6		
DON (mix)	NC	4588	160.6	<LOD	73.9					46 [2]	
ZEN (mix)	NC	919.3	32.2	<LOD	6.0	α -ZEL	<LOD	<LOD	3.8	49 [2]	
						β-ZEL	0.4	<LOD	6.2		
DON (mix)	AC	939	32.9	<LOD	18.5					56 [2]	
ZEN (mix)	AC	427	14.9	<LOD	6.0	α -ZEL	<LOD	<LOD	1.2	75 [2]	
						β-ZEL	0.4	<LOD	4.2		
DON (mix)	AC	2101	73.5	<LOD	39.3					53 [2]	
ZEN (mix)	AC	2283	79.9	<LOD	26.2	α -ZEL	<LOD	<LOD	6.8	62 [2]	
						β-ZEL	1.7	<LOD	17.3		
DON (mix)	S	568	19.9	<LOD	11.5					58 [2]	
ZEN (mix)	S	589	20.6	<LOD	11.6	α -ZEL	<LOD	<LOD	0.3	67 [2]	
						β-ZEL	<LOD	<LOD	2.0		

DON (mix)	S	576	20.2	<LOD	11.7					58 [2]	
ZEN (mix)	S	2254	78.9	<LOD	49.6	α -ZEL	<LOD	<LOD	3.8	79 [2]	
						β-ZEL	0.1	<LOD	9.0		
FB_1	S	50,000	44,000		31,000					38 [1]	Abado Becognee et al. (1997)
	S	150,000	140,000		87,000					42 [1]	
	S	450,000	398,000		276,000					39 [1]	
DON	NC	4,900		<LOD	1,140	DON-3G		<LOD	<LOD	14 [1]	van Broekhoven et al. (2017)
						15-AcDON		<LOD	<LOD		
DON	S	8,000		<LOD	4,980	DON-3G		<LOD	<LOD	41 [1]	
						15-AcDON		<LOD	<LOD		
T-2 (mix)	NC		88.8	<LOD	58.6	HT-2	11.1	<LOD	<LOD	59 [2]	Piacenza et al. (2020)
						T-2 triol		<LOD	<LOD		
						T-2 tetraol		<LOD	<LOD		
T-2 (mix)	NC		262.3	<LOD	135.8	HT-2	26.0	<LOD	<LOD	47 [2]	
						T-2 triol		<LOD	<LOD		

						T-2 tetraol		<LOD	<LOD		
T-2 (mix)	AC		53.9	<LOD	29.7	HT-2	51.6	<LOD	34.1	60 [2]	
						T-2 triol		<LOD	<LOD		
						T-2 tetraol		<LOD	<LOD		
T-2 (mix)	AC		139.8	<LOD	51.1	HT-2	120.9	<LOD	34.1	33 [2]	
						T-2 triol		<LOD	<LOD		
						T-2 tetraol		<LOD	<LOD		
Alphitobius diaperinus											
Parent compound	Type of contamination	Initial Dose (µg/kg)	Initial Conc. in Feed (µg/kg)	Conc. in Larvae (µg/kg)	Conc. in Residue(s) (µg/kg)	Metabolite (s)	Initial Conc. in Feed (µg/kg)	Conc. in Larvae (µg/kg)	Conc. in Residue (µg/kg)	Total Calculated Mass Balance %	Reference
AFB_1	S	20 (ww)	8 (ww)	<LOD	5.7 + <LOQ (dw)	AFM_1		<LOQ	<LOQ	56 [1]	Camenzuli et al. (2018)
						AFL		<LOQ	<LOQ		
						AFP_1		<LOQ	<LOQ		
						AFQ_1		<LOQ	<LOQ		
	S	200 (ww)	70 (ww)	<LOD		AFM_1		<LOQ	2.0 (dw)	79 [1]	

					61.7 + <LOQ (dw)	AFL		<LOQ	<LOQ		
						AFP_1		<LOQ	<LOQ		
						AFQ_1		<LOQ	<LOQ		
	S	500 (ww)	390 (ww)	<LOD	326.7 + <LOQ (dw)	AFM_1		<LOQ	11 (dw)	69 [1]	
						AFL		<LOQ	1.5 (dw)		
						AFP_1		<LOQ	<LOQ		
						AFQ_1		<LOQ	<LOQ		
AFB_1 (Mix)	S	20 (ww)	18 (ww)	<LOD	17.7 + <LOQ (dw)	AFM_1		<LOQ	<LOQ	79 [1]	
						AFL		<LOQ	<LOQ		
						AFP_1		<LOQ	<LOQ		
						AFQ_1		<LOQ	<LOQ		
	S	200 (ww)	180 (ww)	<LOD	153.3 + <LOQ (dw)	AFM_1		<LOQ	5.5 (dw)	71 [1]	
						AFL		<LOQ	<LOQ		
						AFP_1		<LOQ	<LOQ		
						AFQ_1		<LOQ	<LOQ		
	S	500 (ww)	430 (ww)	<LOD		AFM_1		<LOQ	11 (dw)	80 [1]	

					413.3 + <LOQ (dw)	AFL		<LOQ	1.3 (dw)		
						AFP_1		<LOQ	<LOQ		
						AFQ_1		<LOQ	<LOQ		
DON	S	5,000 (ww)	3,900 (ww)	<LOD	4,300 + <LOQ (dw)					90 [1]	
	S	50,000 (ww)	38,000 (ww)	<LOD	44,700 + <LOQ (dw)					96 [1]	
	S	125,000 (ww)	112,000 (ww)	<LOD	110,000 + 263.3					80 [1]	
DON (Mix)	S	5,000 (ww)	4,100 (ww)	<LOD	4,700 + <LOQ (dw)					92 [1]	
	S	50,000 (ww)	41,000 (ww)	<LOD	47,300 + <LOQ (dw)					91 [1]	

	S	125,000 (ww)	100,000 (ww)	<LOD	116,700 + <LOQ (dw)					94 [1]	
OTA	S	100 (ww)	170 (ww)	<LOD	210 + <LOQ (dw)					97 [1]	
	S	1,000 (ww)	1,300 (ww)	<LOD	2,400 + <LOQ (dw)					111 [1]	
	S	2,500 (ww)	1,700 (ww)	<LOD	1,900 + <LOQ (dw)					115 [1]	
OTA (Mix)	S	100 (ww)	80 (ww)	<LOD	120 + <LOQ (dw)					116 [1]	
	S	1,000 (ww)	800 (ww)	<LOD	1,100 + <LOQ (dw)					111 [1]	
	S	2,500 (ww)	2,000 (ww)	<LOD	3,100 + 2.4 (dw)					126 [1]	

ZEN	S	500 (ww)	280 (ww)	<LOD	280 + <LOQ (dw)	α -ZEL		<LOQ	23 (dw)	88 [1]	
	S					β-ZEL		<LOQ	<LOQ		
	S	5,000 (ww)	2,500 (ww)	<LOD	3,000 + <LOQ (dw)	α -ZEL		<LOQ	203 (dw)	109 [1]	
	S					β-ZEL		<LOQ	102 (dw)		
	S	12,500 (ww)	13,000 (ww)	<LOD	13,700 + <LOQ (dw)	α -ZEL		<LOQ	820 (dw)	91 [1]	
	S					β-ZEL		<LOQ	320 (dw)		
ZEN (Mix)	S	500 (ww)	400 (ww)	<LOD	490 + <LOQ (dw)	α -ZEL		<LOQ	30 (dw)	105 [1]	
	S					β-ZEL		<LOQ	11 (dw)		
	S	5,000 (ww)	3,800 (ww)	<LOD	4,300 + <LOQ (dw)	α -ZEL		<LOQ	350 (dw)	100 [1]	
	S					β-ZEL		<LOQ	140 (dw)		
	S	12,500 (ww)	9,400 (ww)	<LOD		α -ZEL		<LOQ	740 (dw)	117 [1]	

	S				12,700 + <LOQ (dw)	β-ZEL		<LOQ	270 (dw)		
DON	NC, CG		1207	726	827	3-AcDON	<LOD	<LOD	<LOD	± 43 [1]	Leni et al. (2019)
FB_1	NC, CG		727	127	728					± 55 [1]	
FB_2	NC, CG		294	<LOD	<LOD					± 6 [1]	
DON	NC, WM		938	416	<LOD	3-AcDON	<LOD	<LOD	<LOD	± 6 [1]	
DON	NC, CDR		779	468	587	3-AcDON	<LOD	<LOD	<LOD	± 22 [1]	
FB_1	NC, CDR		573	<,LOD	224					± 12 [1]	
FB_2	NC, CDR		441	<LOD	<LOD					± 2 [1]	

AC: Artificially Contaminated, CDR: Corn Distillation Residues, CG: Corn Gluten Feed, Conc.: concentration, dw: dry weight, LOD: Limit of Detection, LOQ: Limit of Quantification, NC: Naturally Contaminated, S: Spiked, WM: Wheat Middlings, ww: wet weight.

[1] The percentage of the mass balance is as calculated by the respective authors.

[2] Mass balance = calculated as follows: $\frac{\text{Concentration of Parent Compound and Metabolites in Residues}}{\text{Initial Concnetration of Parent Compound and Metabolites in Feed}} * 100$

[3] Nivalenol is not a metabolite of DON, but a known co-occurring type B-trichothecene, which was not present in the initial diets, but was found back in the residue material.
